# Regulatory T Cells (Tregs) and COVID-19: Unveiling the Mechanisms, and Therapeutic Potentialities with a Special Focus on Long COVID

**DOI:** 10.3390/vaccines11030699

**Published:** 2023-03-19

**Authors:** Manish Dhawan, Ali A. Rabaan, Sara Alwarthan, Mashael Alhajri, Muhammad A. Halwani, Amer Alshengeti, Mustafa A. Najim, Ameen S. S. Alwashmi, Ahmad A. Alshehri, Saleh A. Alshamrani, Bashayer M. AlShehail, Mohammed Garout, Saleh Al-Abdulhadi, Shamsah H. Al-Ahmed, Nanamika Thakur, Geetika Verma

**Affiliations:** 1 Department of Microbiology, Punjab Agricultural University, Ludhiana 141004, India; 2 Trafford College, Altrincham, Manchester WA14 5PQ, UK; 3 Molecular Diagnostic Laboratory, Johns Hopkins Aramco Healthcare, Dhahran 31311, Saudi Arabia; 4 College of Medicine, Alfaisal University, Riyadh 11533, Saudi Arabia; 5 Department of Public Health and Nutrition, The University of Haripur, Haripur 22610, Pakistan; 6 Department of Internal Medicine, College of Medicine, Imam Abdulrahman Bin Faisal University, Dammam 34212, Saudi Arabia; 7 Department of Medical Microbiology, Faculty of Medicine, Al Baha University, Al Baha 4781, Saudi Arabia; 8 Department of Pediatrics, College of Medicine, Taibah University, Al-Madinah 41491, Saudi Arabia; 9 Department of Infection Prevention and Control, Prince Mohammad Bin Abdulaziz Hospital, National Guard Health Affairs, Al-Madinah 41491, Saudi Arabia; 10 Department of Medical Laboratories Technology, College of Applied Medical Sciences, Taibah University, Al-Madinah 41411, Saudi Arabia; 11 Department of Medical Laboratories, College of Applied Medical Sciences, Qassim University, Buraydah 51452, Saudi Arabia; 12 Department of Clinical Laboratory Sciences, College of Applied Medical Sciences, Najran University, Najran 61441, Saudi Arabia; 13 Pharmacy Practice Department, College of Clinical Pharmacy, Imam Abdulrahman Bin Faisal University, Dammam 31441, Saudi Arabia; 14 Department of Community Medicine and Health Care for Pilgrims, Faculty of Medicine, Umm Al-Qura University, Makkah 21955, Saudi Arabia; 15 Department of Medical Laboratory Sciences, College of Applied Medical Sciences, Prince Sattam Bin Abdulaziz University, Riyadh 11942, Saudi Arabia; 16 Dr. Saleh Office for Medical Genetic and Genetic Counseling Services, The House of Expertise, Prince Sattam Bin Abdulaziz University, Dammam 32411, Saudi Arabia; 17 Specialty Paediatric Medicine, Qatif Central Hospital, Qatif 32654, Saudi Arabia; 18 University Institute of Biotechnology, Department of Biotechnology, Chandigarh University, Mohali 140413, India; 19 Department of Experimental Medicine and Biotechnology, Post Graduate Institute of Medical Education and Research (PGIMER), Chandigarh 160012, India

**Keywords:** COVID-19, immune response, SARS-CoV-2, T regulatory cells (Tregs), long COVID, therapeutics

## Abstract

The COVID-19 pandemic has caused havoc all around the world. The causative agent of COVID-19 is the novel form of the coronavirus (CoV) named SARS-CoV-2, which results in immune system disruption, increased inflammation, and acute respiratory distress syndrome (ARDS). T cells have been important components of the immune system, which decide the fate of the COVID-19 disease. Recent studies have reported an important subset of T cells known as regulatory T cells (Tregs), which possess immunosuppressive and immunoregulatory properties and play a crucial role in the prognosis of COVID-19 disease. Recent studies have shown that COVID-19 patients have considerably fewer Tregs than the general population. Such a decrement may have an impact on COVID-19 patients in a number of ways, including diminishing the effect of inflammatory inhibition, creating an inequality in the Treg/Th17 percentage, and raising the chance of respiratory failure. Having fewer Tregs may enhance the likelihood of long COVID development in addition to contributing to the disease’s poor prognosis. Additionally, tissue-resident Tregs provide tissue repair in addition to immunosuppressive and immunoregulatory activities, which may aid in the recovery of COVID-19 patients. The severity of the illness is also linked to abnormalities in the Tregs’ phenotype, such as reduced expression of FoxP3 and other immunosuppressive cytokines, including IL-10 and TGF-beta. Hence, in this review, we summarize the immunosuppressive mechanisms and their possible roles in the prognosis of COVID-19 disease. Furthermore, the perturbations in Tregs have been associated with disease severity. The roles of Tregs are also explained in the long COVID. This review also discusses the potential therapeutic roles of Tregs in the management of patients with COVID-19.

## 1. Introduction

The so-called COVID-19 pandemic, which has caused severe damage to humankind, was caused by the novel form of the coronavirus named SARS-CoV-2. The SARS-CoV-2 infection has shown variability in the prognosis of the COVID-19 disease, which can cause flu-like symptoms, viral pneumonia, multiple organ damage, or acute respiratory distress syndrome (ARDS) [1,2,3,4]. Comparing the SARS-CoV-2 infection to earlier coronavirus infections, one may see unique patterns of cellular and humoral immunological abnormalities [1,4]. In the early and moderate phases, it may cause exhaustion of T cells, dendritic cells (DCs), and natural killer (NK) cells; however, excessive stimulation of such immune cells has been reported in severe instances, leading to a cytokine storm [5]. Cytokine storm has been proposed as the leading cause of death in COVID-19-infected patients [1].

Interestingly, scientists are still unveiling the exact roles of immune cells in deciding the secretion of a balanced number of cytokines and chemokines, which will be essential to elicit the only required immune response despite an exaggerated immune response in the form of uncontrolled release of cytokines and chemokines [4,6]. Numerous researchers hold the view that adaptive immune responses, particularly cell-mediated immune responses, are essential for limiting the SARS-CoV-2 infection by regulating the release of essential cytokines and other anti-inflammatory proteins [6]. Furthermore, the rapid evolution of SARS-CoV-2 into the diverge variants with a plethora of mutations makes scientists think about the cell-mediated immune response seriously [7,8].

Strong T-cell responses have been associated with less severe outcomes in numerous infections. Hyperactivation, however, can potentially have negative effects as the infection spreads [1,9]. Furthermore, in this context, several studies have linked increased levels of effector molecules produced by CD8+ T cells with better clinical outcomes in acute COVID-19 [10,11]. Increased activation of T cells has been linked to a negative outcome of the SARS-CoV-2 infection [12], despite the fact that polyfunctionality peaks in moderate sickness [11]. This suggests that excessive stimulation of immune cells may be deleterious. Virus-specific T-cell responses in asymptomatic infection are characterized by balanced secretion of anti-inflammatory and proinflammatory cytokines such as IL-10 and IL-6 as opposed to symptomatic disease, characterized by more polarized production of inflammatory mediators [13,14,15]. The scientific community is clearly divided on how many activations of immune cells, such as T cells and NK cells, are required. Such contradictory disputes still exist even after multiple advancements in the field of cellular immunology [14,15].

A fine-tuned immune response is vital in determining the outcome of the SARS-CoV-2 infection, and regulatory T cells (Tregs) have been proven to be crucial in controlling the immune response, according to recent research on immune cells. Along with CD4+ and CD8+ T cells, Tregs play a critical role in immunological tolerance and balance [16,17]. Tregs are significant regulators of the inflammatory response. The role of Tregs specific to SARS-CoV-2 in the progression of the illness is currently unknown [17], but systemic inflammation and intense pneumonitis are the major clinical manifestations of severe COVID-19 disease [17,18]. Additionally, virus-specific T-cell responses, especially those of Tregs, have been shown to have an impact on tissue injury in respiratory diseases [18].

Tregs are indeed key subsets of T cells that suppress the immune system. Recent research has shown that COVID-19 patients have significantly fewer Tregs than the general population. Such a decline may have an impact on COVID-19 patients in a number of ways, including diminishing the consequence of inflammatory inhibition, creating an imbalance in the Treg/Th17 ratio, and raising the risk of respiratory failure [13,19]. Treg-targeted treatment may help COVID-19 patients with their symptoms and slow down the disease development [19]. Importantly, it is still not clear whether the decline in Tregs in COVID-19 patients leads to a poor prognosis or whether the increased number of Tregs has beneficial effects. Many scientists believe that a balanced amount of Tregs number is essential to containing any adverse effects of severe infection of SARS-CoV-2 [19,20,21]. Additionally, there are many ways that viral proteins can activate and change T cells. For example, a drop in Foxp3 levels can induce the activation of Tregs or the death of Treg cells. The expression of S-protein on the SARS-CoV-2’s surface is required for the invasion into the host. Furin, a pro-protein convertase, activates the S-protein, and its T-cell-specific deletion activity impairs FoxP3 and TBX21, which induce Treg development. CD4+ T cells are hyperactivated in severe COVID-19 patients, although Foxp3 expression is suppressed. Before developing into Tregs, a significant fraction of T cells get activated, multiply, and expire quickly [19]. However, how S-protein and other viral proteins can lead to the generation of specific Tregs is still unclear.

It is also worth noting that recent research has proposed that the pathophysiology of COVID-19 may be influenced by changes in Tregs, important regulators of immunological homeostasis, but how much change is required to induce a controlled secretion of cytokines is yet to be uncovered. Severely infected patients with COVID-19 have shown unique Treg phenotype and increased expression of its characteristic transcription factor FoxP3 [20,21]. Such Tregs have shown a distinctive transcriptional profile, with upregulation of a number of suppressive effectors as well as proinflammatory molecules, including IL-32, and remarkable similarities to tumor-infiltrating Tregs that inhibit antitumor responses [20]. These characteristics were most obvious during acute, severe illness, and some of them continued in recovering individuals. IL-6 and IL-18 may each contribute various aspects of these COVID-19-linked perturbations, according to a screen for potential agents [20,21]. These findings imply that Tregs may have negative effects on COVID-19 by directly promoting inflammation and inhibiting antiviral T-cell responses during the disease’s acute phase [20,21,22].

Hence, the immunological cells such as Tregs, which are intended to moderate hyperactivated immune responses, should be carefully considered to develop the therapeutic modalities not only against SARS-CoV-2 but also for the other viral infections [1,3,20]. Therefore, this article focuses on the current knowledge of Tregs’ function in the modulation of immune responses to COVID-19. Furthermore, insufficient research has been conducted on regulatory T cells (Tregs) in patients with long COVID and recovering COVID-19 patients [20]. Aspects of Treg function, such as cytokine production or suppressive efficacy in long COVID, have not been investigated in any of the current clinical studies [23]. Due to a dearth of studies addressing Tregs in these aspects, it is hard to draw clear conclusions on the kind of Treg adaptations in long COVID and their potential therapeutic involvement in long COVID management. In this context, we have uncovered recent information on the therapeutic potential of Tregs in the management of COVID-19 and long COVID.

## 2. Immunoregulatory Functions of Tregs

Tregs are required to ensure immunologic homeostasis and self-tolerance and halt exaggerated immunological responses. All these mechanisms are tightly controlled by the balanced expression of the FoxP3 transcription factor. In human peripheral blood, Tregs make up 10% of CD4+ T cells, and these CD4+ cells express specific markers such as FoxP3, which enable their immunosuppressive activities [24,25]. Through a variety of effector pathways, Tregs control the stimulation of several innate and adaptive immune system pathways [20,21]. Additionally, specific “tissue Treg” populations regulate homeostasis in a number of non-immunological tissues, reducing inflammation and encouraging orderly tissue regeneration [20,26,27]. However, Tregs may also be harmful. This is best shown by the fact that they inhibit powerful cytotoxic responses in tumors, where they take on unique phenotypic characteristics [28]. On antiviral responses, they may potentially have contradictory effects [20,29,30], which can lead to a higher viral load.

Previously, it has been reported that the co-transfer of Tregs cells can prevent autoimmune disease in athymic nude mice [31]. Many studies have shown that FOXP3+ Tregs play an important role in maintaining fetal-maternal tolerance, oral tolerance, transplantation tolerance, and mucosal tissue tolerance via various immune suppressive pathways [30,32,33,34,35].

**Figure 1:**
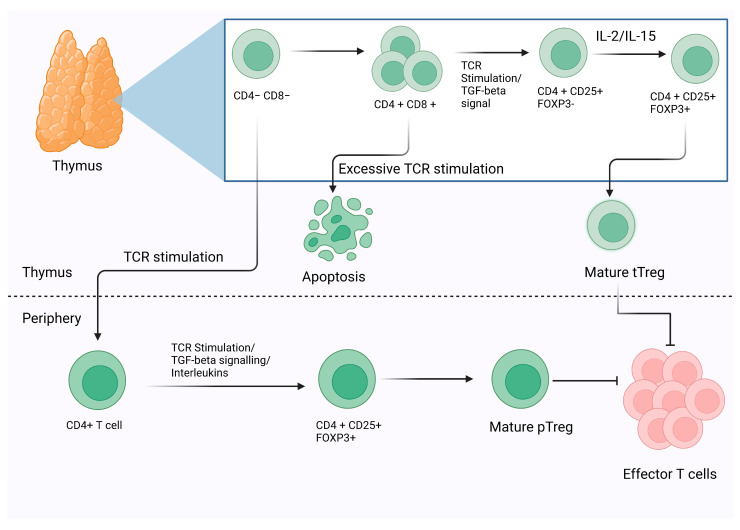
The schematic representation of the morphogenesis and development of Tregs. On the basis of the development and their functional markers, Tregs have been classified into two major categories thymic Tregs (tTregs) and peripheral Tregs (pTregs). Additionally, mature CD4+ Th cells can be induced into Tregs by TCR stimulation.

Before understanding the mechanisms by which Tregs imply their suppression, it is essential to note that Tregs can be majorly divided into two categories, including thymic Tregs (tTregs) and peripheral Tregs (pTregs) based on their site of morphogenesis or development. The tTregs develop in the thymus from precursors of CD4+ helper T (Th) cells, whereas peripheral Tregs (pTregs) differentiate from mature CD4+ Th cells in the periphery. Induced Tregs (iTregs) are a third form of Treg that could be developed ex vivo using mature CD4+ Th cells by stimulating the T-cell receptor (TCR) and by administering TGF-beta [36]. It is commonly acknowledged that the characteristic of Treg morphogenesis in humans is the simultaneous expression of Foxp3 and IL-2 receptor alpha-chain (CD25) with a reduced IL-7 receptor (CD127) expression [37] [Figure 1]. Surprisingly, numerous researchers have shown that a low dosage of IL-2 may increase Treg number and function. Some recent studies have revealed that a low dose of IL-2 may grow autologous Treg cells, which can be used to treat a variety of inflammatory disorders [38].

The mechanisms by which Tregs suppress the immune response or regulate the immunological processes can be divided into active and counteractive mechanisms [27]. The active mode involves the production of immune suppressive cytokines by Tregs, including IL-10, TGF-beta, IL-35, and adenosine. At the same time, the counteractive mode entails the removal of components essential for the activation and survival of effector T cells, including peptide-MHC class II, CD80-CD86, and IL-2 [39,40,41]. Although the exact immunosuppressive mechanisms that operate in vivo are not well understood, it is generally agreed that activation of the Treg TCR occurs before suppressive action [27].

**Figure 2:**
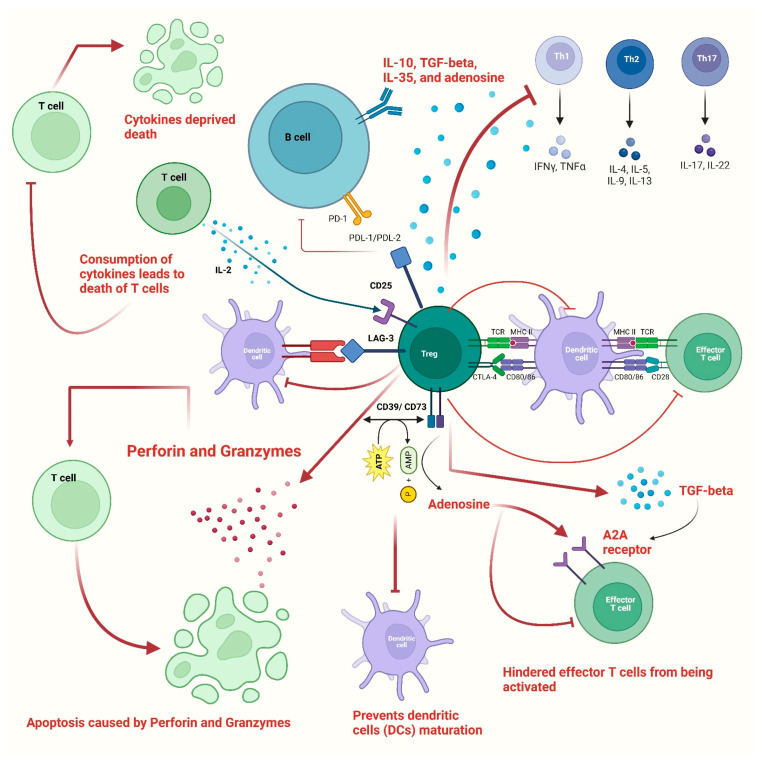
The figure shows various immunosuppressive mechanisms used by Tregs to control the immune system. Cells of both adaptive and innate immune responses are suppressed by Tregs via either direct or indirect mechanisms. Tregs have the ability to generate TGF-beta, IL-10, and IL-35, which have an inhibitory effect on T cells. This can lead to suppressed actions of Th1, Th2, and Th17 type T cells. Due to the strong expression of IL-2 receptors, i.e., CD25, Tregs can cause cytokine-deprived death of effector T cells. Additionally, the lack of IL-2 prevents natural killer cells from multiplying and acting as effector cells. Tregs have been shown to directly affect B cells through the PDL1/PD-1 interaction. Tregs can inhibit the macrophages by increasing CD80/CD86 expression, which gets stimulated through CTLA-4. The proliferation of T effectors is decreased by the expression of CD39 on Tregs, which mediates the conversion of ATP to adenosine and AMP. A2A receptors on T cells get stimulated by AMP and hinder the activation of effector T cells. Additionally, the usage of ATP and its conversion into AMP inhibits the activation of dendritic cells. Moreover, Tregs also produce granzyme and perforin, which damage the T cells’ membrane, which in turn leads to cell death or apoptosis.

Additionally, Tregs express CD39, which helps in the metabolism of ATP to AMP, which in turn prevents dendritic cells (DCs) maturation due to the depletion of ATP [42]. Furthermore, co-expressed CD39 and CD73 on Tregs were able to convert ADP into adenosine, which coupled to the effector T cell’s adenosine A2A receptor and hindered effector T cells from being activated [43,44]. Consequently, by decreasing the expression of IL-6 and increasing the synthesis of TGF-beta, the stimulation of the adenosine A2A receptor encouraged the development of Tregs [45]. Furthermore, Tregs may suppress the expression of the costimulatory molecules CD80 and CD86 on DCs [20,43]. Antigen-presenting cells (APCs) were unable to get activated as a result of Tregs’ production of CTLA-4, which decreased CD86 via transendocytosis [44,46]. Additionally, by increasing the expression of indoleamine 2,3-dioxygenase in DCs through CTLA-4-induced signaling, Tregs might starve effector T cells [47,48], which in turn suppresses the immune response [Figure 2].

Aside from that, Tregs produced the lymphocyte activation gene 3 (LAG-3), which competitively bonded to the major histocompatibility complex class II (MHC-II) and prevented dendritic cells (DCs) from maturing [49]. The cytolysis of CD8+ T cells and NK cells by Granzymes- and Perforin-dependent means constituted another significant Treg-mediated suppressive pathway [41]. According to Gotot et al. (2012), in addition to apoptosis caused by Perforin and Granzymes, programmed death-ligand 1(PD-L1) of Tregs and programmed death-1 (PD-1) of autoreactive B lymphocytes interfere with the proliferation and functionality of autoreactive B lymphocytes [50] [Figure 2].

## 3. Possible Roles of Tregs in COVID-19 Pathogenesis and Disease Severity

While the pandemic was at its peak, numerous studies have discussed the possible connection between Treg and the severity of COVID-19. According to some studies, the percentage of Tregs is rising, or their functional markers are being expressed more strongly in severely infected patients with COVID-19 [51,52]. For instance, one recent research found that severe COVID-19 patients had greater levels of CD25+ FOXP3+ Tregs among CD4+ T cells, increased FOXP3 expression, and elevated production of activated Treg markers including KLRG1 and PD-1, all of which returned to normal levels in the recovering individuals or convalescent patients [51]. Likewise, another recent observation found that the number, multiplication, and expression of certain proteins of CD25+ CD127+ FOXP3+ Tregs increased, along with their growing suppressive activity, in severely infected patients with COVID-19 [52]. Scientists discovered increased Tregs and Th17 cells as well as decreased T-cell numbers in the bronchoalveolar lavage fluid (BALF) of COVID-19 patients with ARDS [53].

**Figure 3:**
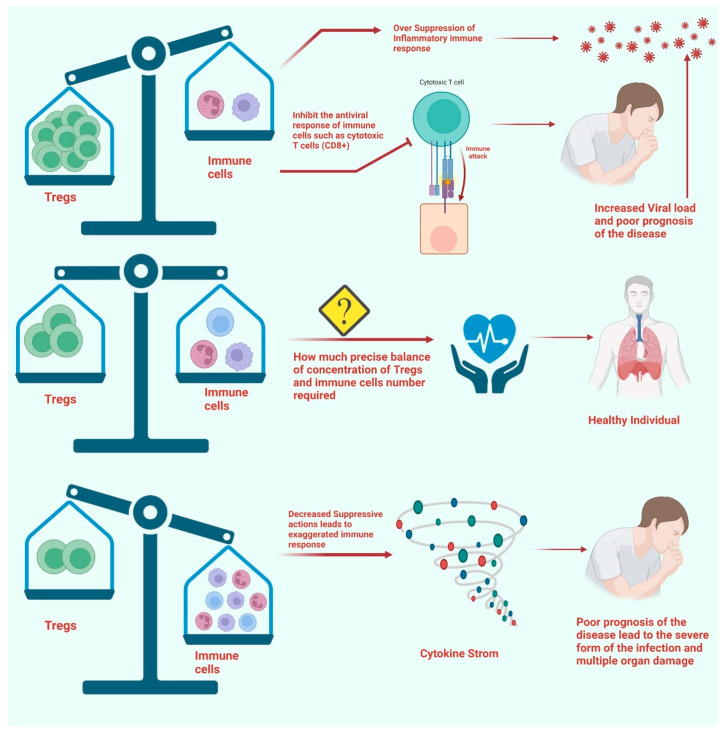
The figure represents the Treg involvement in the pathophysiology of COVID-19. The increased number of Tregs in severely infected patients can play deleterious effects by limiting the antiviral effects of effector T cells. Additionally, the overly expressed FoxP3 in Tregs can lead to excessive immunosuppressive activities, which lead to a poor prognosis of the disease. On the other hand, the substantial decrease in the number of Tregs cannot alleviate the excessively stimulated immune response in severely infected patients. Moreover, a balanced number of Tregs compared to Th1/Th17 T cells and other immune cells can prevent the poor prognosis of the disease.

In contrast to the healthy donor population and convalescent patients, the percentage of CD25+ CD127− Tregs among all CD4+ T cells was found to increase significantly in patients with persistent SARS-CoV-2 infection. Additionally, the increased expression of CTLA-4 on Tregs was reported in patients with persistent antigen expression [54]. Intriguingly, most of the patients have been reported to have an increased number of naive Tregs (CD45RA+ CCR7+). Additionally, central memory Tregs (CD45RA− CCR7+) with strong expression of PD-1 were reported in the patients with COVID-19 [55]. Additionally, the CD4+ FOXP3+ Tregs of the lung and PBMC showed a rising trend on day five after infection in the nonhuman model of COVID-19 pathogenesis [56]. There has not been much research done on the composition of T-cell subsets in SARS-CoV-2-infected convalescent patients. Based on the number of days following RT-PCR confirmation of SARS-Co-V2 infection, researchers calculated the lymphocyte absolute numbers, the frequency of memory T-cell subsets, and the plasma levels of common gamma-chain in seven groups of COVID-19 patients. The findings demonstrate that CD4+ naive T cells, regulatory T cells, transitional memory, and stem cell memory T-cell frequencies decreased from Days 15–30 to Days 61–90 and then remained steady. Conversely, CD4+ naive, transitional, and stem cell memory T-cell frequencies declined from Days 15–30 to Days 61–90 and then decreased again. Patients with severe COVID-19 had reduced lymphocyte numbers and frequency levels; greater naive cells (Tregs); lower frequencies of central memory, effector memory, and stem cell memory, and higher plasma levels of IL2, IL7, IL15, and IL21. As a result, the research suggests that convalescent COVID-19 people had altered memory T-cell subset frequencies, which will be clarified in the future [57]. Further investigations are needed to confirm this idea, but it is possible that an increase in the cell percentage and the number of functional indicators would result in higher Treg suppression, which can be detrimental to COVID-19 patients [54,56] [Figure 3].

Considering the biphasic functions of Tregs throughout the SARS-CoV-2 infection, it is still debatable how the fraction of Tregs in COVID-19 changed. The number of Tregs has decreased in COVID-19 patients, according to many researchers. For instance, one study found that the Th17/Treg ratio was substantially enhanced while the number of Tregs in ICU-hospitalized patients was reduced significantly [58]. It is interesting to note that the immunomodulatory and immunosuppressive functions of Tregs isolated from severely infected COVID-19 patients were found to be compromised. Another study found a comparable rise in the Th17/Treg ratio in the PBMC of COVID-19 patients, which was associated with a negative outcome and lower levels of TGF-beta and IL-10, cytokines that are important for Tregs [59] [Figure 3]. Additionally, a single-cell transcriptomic evaluation of viral antigen-reactive CD4+ T cells of patients with SARS-CoV-2 infection found that the percentages of SARS-CoV-2-reactive Tregs, T follicular helper cells (Tfh), and cytotoxic T helper cells responsive to the viral infection were enhanced in hospitalized COVID-19 patients [60].

Another comparative analysis reported a reduced number of Tregs in severely infected patients with SARS-CoV-2 as compared to the patients with mild symptoms. It has been concluded that the proportion of Tregs was negatively correlated with viral load, indicating that lower Treg levels can be associated with a higher risk of illness, especially in hospitalized patients with COVID-19 [61]. According to another study, individuals with severe COVID-19 had a lower percentage of regulatory T cells (CD3+ CD25+) [62]. Another research that evaluated the PBMCs of COVID-19 patients found that the Tregs ratio increased as the disease progressed from moderate to severe but decreased as it progressed to critical [63]. This suggests that the Tregs underwent a dynamic shift as COVID-19 progressed.

Intriguingly, a study that examined the gene expression patterns of CD4+ T cells of patients with SARS-CoV-2 infection discovered that CD25 was significantly upregulated [63,64]. It is quite interesting to observe that the Tregs of severely infected patients reported having a reduced level of master transcription factor (FOXP3+). The increased level of FURIN appeared to be related to the increased level of CD25, which may facilitate SARS-CoV-2 entry into lung epithelial cells and reduce the immunosuppressive activities of Tregs in patients with poor disease prognoses. Tregs seem to have decreased during the acute phase of the SARS-CoV-2 infection in children and returned to baseline after recovery [64]. Surprisingly, a high-dimensional flow cytometry examination of the airways of severely infected COVID-19 patients revealed decreased Treg frequency as compared to healthy individuals [65]. This finding raises the possibility that the functionality of tissue-resident Tregs, especially residential to lungs, was compromised in severely infected patients with COVID-19. It is well-established that pro-inflammatory cytokines such as IL-6 may cause Tregs to lose their stability in vitro [66]. Therefore, under the inflammatory conditions brought on by COVID-19, high levels of IL-1, IL-6, and IL-23 may promote the downregulation of FOXP3 [67], resulting in decreased functionality of Tregs.

Additionally, it was shown that COVID-19 patients’ Treg subset composition varied. For instance, the research found that in adult patients with more severe illnesses, the ratio of CD39+ Tregs in PBMCs increased, but in young patients, the ratio of CD39+ Tregs dropped in an age-dependent way [68]. According to another research, only CCR4Hi Tregs in hospitalized COVID-19 patients elevated, while total Tregs remained unchanged [69]. According to Chen et al. (2020), when compared to moderately infected COVID-19 patients, the severely infected subjects demonstrated a substantially decreased proportion of CD45RA+ memory Tregs and a fractionally greater percentage of CD45RO+ naive Tregs, implying that the proportion of Treg subsets may be able to anticipate the prognosis of the disease cases [70]. Other research found a similar pattern, particularly in severely infected patients with COVID-19, as compared to moderately infected ones [20,21].

According to reports, individuals who had COVID-19 and lymphopenia had a worse prognosis; lower blood lymphocyte percentages suggested this [71]. Multiple possible processes might be at work in lymphopenia. Additionally, via single-cell RNA-sequencing, the SARS-CoV-2 RNA was also found in immune cells [72], and it has been postulated that SARS-CoV-2 may have the capability of infecting Treg through ACE2-independent receptors [30,73]. This can substantially affect the functionality of Tregs, which needs to uncover in future studies.

Numerous studies have shown an increase in the percentage or quantity of Tregs in COVID-19 patients (particularly those with the milder condition), but they have also found a decrease in the amount of Tregs in the individuals. For instance, it has been shown that severe COVID-19 patients had significantly fewer Tregs (CD3+ CD4+ CD25hi CD127lo FoxP3+) in their PBMCs [60,74,75]. Single-cell research revealed that FoxP3 expression was noticeably lower in severe COVID-19 patients, despite greater expression of CD25 [76]. In PBMC generated from COVID-19 patients receiving ICU care, recent research looked at Tregs and discovered a sharp fall in the proportion of Tregs along with lower production of FoxP3 and inhibitory cytokines such as IL-10 and TGF-beta [59] [Figure 3].

Furthermore, Mohebbi et al. (2020) also reported the reduced expression of important markers such as CD25 and FOXP 3 in Tregs of severely infected patients with COVID-19 as compared to healthy donors [75]. It is important to consider that following SARS-CoV-2 infection, the number of Tregs (CD3+ CD4+ CD25+) considerably decreased throughout the development of infection and symptoms [21]. Scientists are still figuring out the reasons behind the increase in the number of Tregs in moderate conditions of infection and then rapidly decreased as the infection progressed. In adults and children with severe COVID-19, the fraction of Tregs was observed to be lower in other studies as well [60,64]. Such data suggest that the excessive inflammation and pathophysiology of COVID-19 may be responsible for a decreased number of Tregs as well as enhanced Th17 responses. Additionally, recent research found that patients with COVID-19 had higher proportions of Tregs (characterized by the presence of CD3+ CD4+ CD25+ markers) and higher levels of FoxP3 expression by Tregs, which were associated with a poor prognosis of the disease [20]. Such Tregs are reported to secrete a range of immunosuppressive molecules along with inflammatory cytokines such as IL-32, which in turn can limit anti-viral T-cell responses, simultaneously increasing inflammatory responses in severely infected patients with COVID-19 [20,21].

It is important to note that certain investigations, such as those in cancer patients infected with SARS-CoV-2 [77], did not notice any variation in the number of Tregs in the peripheral blood of COVID-19 patients [78]. Additionally, many other studies could not conclude the exact change in the number of Tregs in severely and moderately infected patients with SARS-CoV-2 [20,21]. As a result, there is still debate around recent data on the absolute and relative numbers of Treg cells in COVID-19 patients [21]. This is likely due to the various parameters employed to identify Tregs and the fact that the observation was taken at various phases of the illness [21,22].

Moreover, the patients with reduced or less amount of Tregs along with the low concentration of master regulator FoxP3 were reported to have less severe outcomes of the SARS-CoV-2 infection. Importantly, there are possibilities that these Tregs are advantageous, especially in regulating the cytokine storm that can be severe without the immunosuppressive activities of such Tregs [21]. Nevertheless, insufficient cell numbers made it impossible to directly evaluate their immunosuppression capabilities [79]. Contradictorily, the increased number of Tregs and increased expression of FoxP3 and other effector molecules can interfere with the antiviral response of immune cells such as cytotoxic T cells (CD8+) in the severe phase of the infection [80] [Figure 3] as compared to the initial phase of the infection which in turn can lead to the secondary re-expansion of disease [20,79,81]. However, the exact reasons behind such a shift are yet to be resolved clearly. However, this can be associated with enhanced levels of FoxP3 and other Treg effector molecules along with phenotypic similarities with the immunosuppressive tumor Tregs [20,21].

It is also quite fascinating since the wide community of scientists suggests that Tregs during the SARS-CoV-2 infection get activated, suggesting their immunoregulatory or immunosuppressive activities to prevent immune cells of both innate and adaptive immune response from damaging self-tissues mainly by limiting the excessive release of pro-inflammatory cytokines and chemokines [20,82]. However, it is also possible that in the early stages of infection, a greater proportion of activated Tregs might weaken the immune system’s ability to fight off viruses such as SARS-CoV-2 [9,20]. The excessive production of pro-inflammatory cytokines that causes ARDS, however, may be caused by a decrease in the number of Tregs with compromised functions in severe instances or later stages of the illness [21]. However, a significant amount of research has been done in this field; these ambiguities are yet to be resolved with non-human or human models to understand the immunological response to SARS-CoV-2.

## 4. Perturbations in Tregs and Disease Severity

A significant number of variations in the phenotypic characteristics of Tregs have been reported in severely infected patients with COVID-19, along with increased FoxP3 expression with a unique transcriptional pattern that is very similar to tumor Tregs [83,84]. A broad range of transcriptional patterns have been observed in Tregs, which includes an elevation of interferon-stimulated genes, and these changes have been reported in various other viral infections. However, unusual cell proliferation and heightened effector functions, including ENTPD1, LAG3, and LRRC32, have been observed in Tregs of severely infected patients with COVID-19. Among many of the increased “Severe COVID-19 Treg Signature” (SCTS) transcripts are several members of the tumor necrosis factor (TNF) receptor family, which play crucial roles in Treg function and homeostasis [84,85]. Additionally, increased expression of CXCR3 has been observed in the Tregs of severely infected patients with COVID-19 [85]. CXCR3 receptor for binds to CXCL10 chemokine (member of the CXC chemokine family) to exert its biological effects. CXCL10 has been shown to be a key biological marker modulating illness severity and may be used as a prognostic marker for a number of disorders [86].

Recent findings raise two very important questions. First, where do such perturbations/variations come from? They are not brought on by infecting Tregs with viral particles. Since none of the treatments given to these individuals are correlated with the Treg features, they seem to be unrelated to therapeutic interventions [20,87]. The immunologic environment in such individuals is more likely to be the cause of the phenotypic changes, which is unique to Tregs since Tconvs are much less labeled [87,88]. Additionally, TCR-mediated stimulation is unlike to stimulate the phenotypic changes, given the extensive impact on Tregs in the single-cell data [88], which presumably transcends clonotypic specificity, and the significant loss of Nur77 (NR4A1) [20].

Previous findings indicate that a number of variables are involved, namely IL-6 and IL-18 (although other variables may also be involved), each of which contributes to a different feature of the disturbed Treg phenotype. Since IL-6 is often considered a Treg antagonist and prevents FoxP3 expression by TGF-beta/IL-2 in culture, its effect in this situation first seems counterintuitive [89]. Recent research has provided a more nuanced picture of the role of IL-6 in Treg cells, showing that it is necessary for the development of the RORγ+ Treg subsets and may enhance their suppressive properties [90,91,92]. Tregs with effective inhibitory functionality is considerably more prevalent in transgenic mice with persistently higher serum IL-6 concentrations [93], as the recent findings found perturbations in Tregs of rheumatoid arthritis patients, which might play an important role in the clinical manifestation of the disease [94]. Hence, direct evaluation of Tregs from the lungs of COVID-19 patients would have been beneficial.

Additionally, there are other cytokines, such as IL-18, which determine the phenotypic and functional characteristics of Tregs. IL-18 signaling from epithelial cells to Tregs is necessary for defense against colitis in the RAG transfer paradigm, and it has been shown that IL-18 promotes Treg reparative activity through amphiregulin [95,96]. Recent research suggests that Notch4 and IL-18 signaling work together to regulate pro-reparative effects in Tregs [97]. A subfraction of Tregs with a preference for thymus-homing exhibits the IL-18 receptor predominantly [98]. Recent research suggests that IL-18 has a larger influence on Treg cells than only pro-reparative pathways, including a greater range of Treg effector activities that are represented in module M5 (typical Treg transcripts, such as TNFRSF18 or LRRC32) [20]. Additionally, circulating Tregs from patients with severe COVID-19 exhibit decreased amphiregulin expression, suggesting that some of IL-18’s effects may be mitigated by other COVID-19 cytokine storm components [20,21].

Second, it is important to note that, these abnormal Tregs contribute to the physiopathology of COVID-19. Patients who had lower levels of FoxP3, fewer Tregs, and less severe SCTS did better, which brings up the traditional problem of inferring causality from the association. However, it might be possible that these Tregs are advantageous, regulating a cytokine storm that would not have been as bad without their extraordinary contribution. In context to this, a recent investigation on CD8+ T cells from the same patients revealed a lack of SARS-CoV2-reactive cells in the blood throughout the acute stage, which supports this theory [51,99]. However, in the absence of FoxP3 expression, Tregs have been shown to have pro-inflammatory properties [100], which can be detrimental. Hence, it is difficult to conclude the exact roles of Tregs’ perturbations during the SARS-CoV-2 infection.

Asymptomatic COVID-19 patients, along with controls, were examined in a recent study for the expression levels of CTLA4 on the Tregs. CTLA4 is an important immunosuppressive activity marker. The research found that CD45RA+FoxP3+ resting Tregs, activated Tregs, and total Tregs dynamics all were identical [101]. A further investigation examined the surface expression of the Treg inhibitory marker CD127. When compared to healthy donors, they discovered that the expression of CD127 was considerably downregulated in both moderately and severely infected patients with COVID-19. Severely infected patients who recovered afterward were reported to have decreased levels of CD127 on Tregs [70].

Recently, Benamar et al. (2023) have brought attention to the fact that Multisystem Inflammatory Syndrome in Children (MIS-C) develops in certain pediatric patients after acute infection with SARS-CoV-2 via unidentified pathways. They have demonstrated that Tregs in MIS-C were destabilized through a Notch1-dependent pathway, while acute COVID-19 severity and outcomes were previously associated with Notch4 expression on Tregs. Due to dominant-negative mutations in the Notch1 regulators NUMB and NUMBL, which result in Notch1 overexpression, patients with MIS-C displayed enrichment of uncommon detrimental variations impacting the inflammatory and autoimmune pathways, according to genetic analyses [102]. Tregs that had been stimulated by Notch1 signaling produced CD22, which was then destabilized by mTORC1 and promoted systemic inflammation. These findings suggest unique immunological checkpoints regulated by individual Treg Notch receptors that influence the inflammatory outcome in SARS-CoV-2 infection and reveal a Notch1/CD22 signaling pathway that affects Treg function in MIS-C [102]. These studies suggest that additional studies are required to uncover the vast number of variations/perturbations in the Tregs of severely infected patients with COVID-19.

## 5. Tregs Association with Long COVID

According to Guan et al. (2020), the clinical manifestations of SARS-CoV-2 infection vary from asymptomatic/mild illness to severe pneumonia and respiratory distress syndrome, which may eventually result in death [103]. It has been noted that COVID-19 could encompass multi-system comorbidities, such as thrombotic events, vasculitis, and myocarditis, despite the fact that most patients only perceive mild symptoms such as fever, sore throat, breathing difficulties, loss of smell and taste, or cough [104,105].

Apart from the above-mentioned manifestations of the COVID-19 disease, severe immunopathology has been considered an important characteristic in various cases. The poor prognosis of the SARS-CoV-2 infection or the multiple organ damage is specifically associated with defective T-cell-mediated immune response, which is characterized by excessive proinflammatory cytokines, reduced number of lymphocytes, and newly developed or worsened autoimmune response [23,106]. According to a recent observational cohort research, one in eight individuals who caught COVID-19 is thought to have symptoms that last longer than the acute symptomatic period [107]. The World Health Organization (WHO) describes these sequelae, also known as “long COVID” in common usage today, as a post-COVID-19 condition that typically manifests three months following a confirmed or suspected SARS-CoV-2 infection and includes a group of new-onset, prolonged, or varying ailments that persists for at least two months [108]. According to several studies, the most prevalent symptoms in this situation include exhaustion, breathlessness, post-exertional malaise, chronic cough, headache, muscle aches, tachycardia, concentration deficits, and a decreased quality of life [23,109,110,111].

Numerous possibilities are now being discussed; however, it is still unclear what pathophysiological pathways contribute to the emergence and progression of long COVID. Moreover, Merad et al. (2022) proposed that immunopathological mechanisms, including systemic inflammation with viral persistence, post-viral autoimmune response, microbiome dysbiosis, and unrepaired tissue damage, may be involved in the pathogenesis of long COVID [112]. Accordingly, it has been shown that impacted individuals show a considerable increase in the number of inflammatory markers when compared to those who have recovered, suggesting a hyperactive and disrupted immune response, especially consisting of T cells [113,114]. But the interesting fact is that only a few studies have been done on the involvement of Tregs in patients suffering from long COVID or recovering patients from long COVID. Tregs can be important components of the immune system that may control and fine-tune autoimmune responses, promoting immunological homeostasis in the long COVID [23].

Recent investigations examined the percentage of Tregs among CD4+ cells in patients who still have COVID-19 symptoms and contrasted these to seronegative controls and COVID-19 survivors [115,116,117]. The patients who were part of the long COVID group reported a wide range of symptoms, including headaches, palpitations, insomnia, myalgia, fatigue, and shortness of breath. It has been postulated that Tregs can play important roles in the progression of long COVID. Contradictory findings were seen in the two trials that examined individuals who had persistent symptoms almost a year after the illness. A recent study found that patients with long COVID have more than two times the number of Tregs as compared to the fully recovered subjects from COVID-19 [115]. At the same time, a contradictory observation has recorded a considerable decrease in Tregs concentration in patients with long COVID [117]. The proportions of Tregs expressing FoxP3 were recorded in more than 100 patients with long COVID and found to have a reduced number of Tregs compared to the seronegative controls [116]. However, apart from the change in number, none of these studies investigated the immunosuppressive activities of Treg cells.

The above studies that collected various samples of blood while recovering from COVID-19 provide additional evidence for the aforementioned; they found that the intensity of Tregs displayed by subjects at the second and third follow-ups seemed to be comparable to the frequency of seronegative subjects than it was during the first analysis [118,119,120]. In addition, FoxP3 expression was upregulated in the Tregs obtained from the recovering patients [58]. Although the recruited participants recovered from asymptomatic infections, which may have initially caused less severe Treg alterations, as suggested by other studies [19,23], the relevance of this discovery is still unclear. Accordingly, convalescent patients did not exhibit considerably different Treg levels from acute non-hospitalized subjects [20,121].

The lack of research examining Tregs in a particular cohort makes it impossible to draw any definitive inferences on the kind of Treg adaptations in long COVID [23]. Nevertheless, these investigated analyses revealed that Treg dysregulations/perturbations persist in long COVID patients even years after their original SARS-CoV-2 infection [115]. Furthermore, other comparative studies found a higher and lower proportion of Tregs among CD4+ cells in patients with residual symptoms compared to recovered subjects [23]. The number of cells was found to be comparable in terms of the time elapsed from disease onset to follow-up sampling [23,115].

It is important to keep this in mind while understanding such findings because long COVID is a heterogeneous and multidimensional condition with a variety of clinical signs [122]. Previous studies have hypothesized that long COVID may include a number of symptoms and various sub-diagnoses, many of which have no immunological basis [123,124,125]. It is plausible that Treg dysregulation leads to a long COVID-associated immunopathology in numerous ways, as a particular pathophysiological mechanism cannot explain the post-acute consequences of COVID-19 [23,125]. Further, it becomes increasingly clear that long COVID is not a single diagnosis but rather a group of illnesses with various pathophysiology components. Therefore, it may be considered that the abovementioned studies looking at long COVID depicted a mix of distinct illnesses considering the variety of symptoms shown by the recruited individuals [20,23]. Additional research should explore whether Treg frequencies and the Th17/Treg ratio are correlated with certain symptom combinations, as previously suggested, or with laboratory variables like immunological status, cytokine levels, or autoantibody titers [23].

## 6. Tregs-Based Therapies and Their Therapeutic Potentials

**Figure 4:**
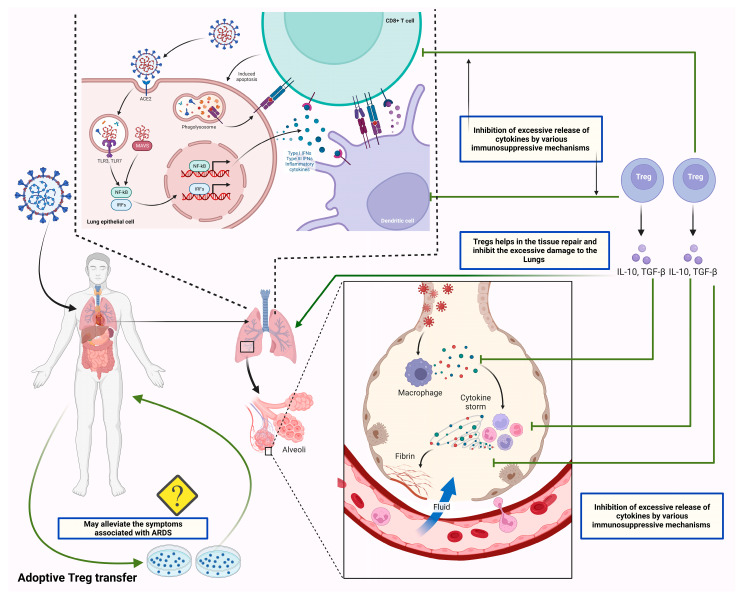
The figure shows the therapeutic potential of Tregs by suppressing the exaggerated immune response. SARS-CoV-2 infects the lung epithelial cells by entering through ACE2 receptors. SARS-CoV-2 infects lung epithelial cells by using ACE2 receptors. As viral RNA enters the cell, it activates endosomal and cytoplasmic sensors, including TLR3/7 and MAVS. Further, these endosomal and cytoplasmic receptors activate IRFs and NFkB, resulting in the production of inflammatory cytokines such as interferons (IFN). Dendritic cells acquire antigen before migrating to lymphoid organs to activate adaptive immunity. Following the recognition of antigens on antigen-presenting cells (APCs) or infected cells, CD8 T lymphocytes trigger apoptosis. Additionally, the viral antigens present to the T cells through antigen processing. Antigen processing is the process through which APCs, such as dendritic cells and alveolar macrophages, endocytose and kill the SARS-CoV-2 virus. MHC proteins then express antigen fragments on the cell membrane, enabling T lymphocytes to identify them. The overstimulated T cells and APCs in severely infected patients lead to excessive secretion of cytokines/chemokines, which leads to lung damage and ARDS. The balanced concentration of Tregs can suppress the exaggerated immune response through its immunoregulatory and immunosuppressive activities. In this context, recent studies suggested the beneficial effects of the adoptive transfer of Tregs in severely infected patients, which is yet to be approved for its clinical safety.

Many studies have implied that Tregs play a protective role by regulating the exaggerated immune response reported in severely infected patients with COVID-19. The deleterious consequences associated with the uncontrolled release of cytokines in severely infected patients with COVID-19 may be managed by using Tregs’ immunosuppressive capabilities [21] [Figure 4].

A potential cellular therapeutic approach for the management of autoimmune disorders and graft-versus-host disease is adoptive Treg transfer [126]. It is crucial to note that earlier research has shown the efficacy of such an intervention in several preclinical ARDS models [127]. Additionally, according to mouse models, the transfer of Tregs may increase the chance that a mouse infected with coronavirus-induced encephalitis would survive and decrease the amount of cardiac fibrosis that the virus causes [128]. Ex vivo expanded Treg cells may therefore be able to restore Treg balance in patients with decreased Treg activity brought along by SARS-CoV-2 infection and subsequently lessen the severity of life-threatening symptoms by reducing excessive inflammation and suppressing the uncontrolled release of cytokines/chemokines [21] [Figure 4].

The effectiveness of the adoptive transfer of Tregs derived from allogeneic HLA-matched umbilical cord was reported, which provided directions for the implementation of the adoptive transfer of Tregs to manage COVID-19 [129]. In this research, intravenous allogeneic cord blood-derived Tregs were given to two severely infected patients having ARDS. The patients were given tocilizumab and vasopressors, while only the first patient received hydroxychloroquine and broad-spectrum antibiotics before receiving therapy with Tregs [21,128,129]. The circumstances of both patients significantly improved after two rounds of cell infusion, which was associated with lower levels of proinflammatory cytokines such as IL-6, TNF-alpha, IFN-gamma, IL-8, IL-12, and MCP-4 in the blood. Interestingly, there were no symptoms of an infusion reaction, a resurgence of inflammatory response, or any additional negative repercussions [129].

Hence, the infusion of Tregs derived from cord blood can be an effective therapeutic intervention to manage severely infected patients with COVID-19. Another current clinical study (NCT04468971) is evaluating the effectiveness and safety of Tregs usage in the management of COVID-19 patients [130]. Additionally, hybrid Tregs having characteristics of TREG/Th2 cells are being investigated in another clinical investigation to alleviate the inflammatory response in severely infected patients showing the symptoms of multiple organ damage [130]. These hybrid cells have shown the capability to reduce the inflammatory response and mediate a beneficial impact on respiratory tissues [130,131].

Circulating soluble IL-2 receptor concentrations have been shown to be higher in severely infected patients with COVID-19 [131,132]. By lowering the bioavailability of IL-2 to Treg cells, soluble IL-2 receptors may limit the development of Treg cells in COVID-19 patients [131]. Previous research has shown that in vivo administration of low-dose IL-2 was able to precisely stimulate Treg expansions in patients with type 1 diabetes and graft-versus-host disease [133,134]. Such investigations led to the registration of a clinical trial to examine the effectiveness of low-dose IL-2 in the management of COVID-19 patients with ARDS [124]. Additionally, a recent study reported that the administration of recombinant IL-2 (rIL-2) might significantly enhance the frequency of lymphocytes, including Tregs, in the peripheral blood [135]. Additionally, after receiving rIL-2 therapy, the concentration of CRP dropped [135], which may contain the deleterious consequences of severe viral infection.

Additionally, a phase 3 clinical research (NCT04724629) investigating the effectiveness and durability of treatment using IL-2 or an inhibitor of IL-17 has just been filed for patients with COVID-19. Nevertheless, clinical observation revealed that patients with severe and critical conditions had higher levels of soluble IL-2R. This led to the development of soluble IL-2R as a biomarker for early detection of severe COVID-19 and for estimating clinical progression [30,136,137]. An elevated concentration of soluble IL-2R may be able to scavenge IL-2, indicating that low-dose IL-2 therapy was not the best course of action for treating COVID-19 [138]. According to reports, an anti-human IL-2 (hIL-2) antibody may change the ratio of effector T cells (Teff) to regulatory T cells (Treg) when it is attached to hIL-2 [139].

In a mouse model of experimental autoimmune encephalomyelitis (EAE), the IL-2 monoclonal antibody JES6-1 selectively expanded Tregs and reduced inflammation [140]. Autoimmune disorders and inflammatory conditions are caused by an imbalance of Tregs vs. other immune cells in living organisms. A JAK 1/2 inhibitor called ruxolitinib has been shown to boost FOXP3 abundance and Treg frequency while decreasing Th17 frequency [141]. Ruxolitinib therapy in the Phase I/II study for COVID-19 was finished in 2021. According to recent research, the transitory breakdown of Treg tolerance may stimulate DCs and cause them to produce an effective adaptive immune response against SARS-CoV-2 [142]. Therefore, balancing Tregs and DCs may be a potential approach for treating COVID-19 [30].

Additionally, several small-molecule-based medications that potentially enhance Tregs activity may be employed to stave off the cytokine storm brought on by severe viral infection. Recently, it has been discovered that the GSK3 inhibitor SB216763 might improve the suppressive effect of hiTregs by increasing the release of IL-10 and reducing proinflammatory iTregs [143]. Another research proposed GSK3 inhibition as a viable treatment strategy against SARS-CoV-2 [144]. Tregs formation required the PI3K-Akt-mTOR signaling pathway [145]. In the treatment of Type 1 diabetic patients, rapamycin, an inhibitor of mTOR, promotes the development of Tregs, simultaneously inhibiting the growth of effector T cells [146,147]. In this context, rapamycin therapy has the ability to stop the excessive release of cytokines/chemokines in severely infected patients with COVID-19 [148].

Furthermore, a metabolite of vitamin A called all-trans retinoic acid (atRA) has been shown to decrease the de novo production of Th17 from naive CD4+ T cells and to promote the development of Tregs from naive CD4+ T cells [149,150]. AtRA might preserve the stability and functionality of nTregs in an inflammatory environment in addition to controlling the balance of Tregs/Th17 [151]. By suppressing 3CLpro activity, AtRA was also found to have antiviral effects against SARS-CoV-2 [152].

Treg-based treatment has the possibility of using antigen-specific TCR, which might be guided in the direction of a desired antigen [153]. In animal models of Type 1 diabetes, arthritis, and transplantation, TCR-Tregs could be grown ex vivo and performed better than polyclonal Tregs [154,155,156]. TCR-Treg treatment may offer benefits of a lower dose but greater effectiveness and has therapeutic benefits in COVID-19 patients by attacking a distinct antigen of SARS-CoV-2 [30]. Another tactic is the use of CAR-Tregs, which may attach to tissue-specific self-antigens and focus on suppressive activities on the location of the illness [157]. In several preclinical models, such as experimental autoimmune encephalomyelitis and experimental allergic asthma, CAR-Treg treatment has been shown to be effective [158,159,160]. CAR-Treg treatment for renal transplantation is currently the subject of Phase I/II investigation. Despite receiving a lot of interest in treating tumors and a number of autoimmune illnesses, COVID-19 has not yet been treated with CAR-Treg therapy. Potential uses for CAR-Treg in the treatment of SARS-CoV-2 are made possible by its capacity to generate immunological tolerance [30].

Furthermore, CTLA-4, which is an important functional marker of Tregs, has been studied to manage COVID-19. The stimulatory receptor CD28’s ligands CD80 and CD86 interact with CTLA-4 and lessen co-stimulatory signals for T-cells via boosting trans-endocytosis and the degradation of two ligands [46]. Abatacept, a recombinant Fc-fused form of CTLA-4 protein, has been used for many years as an immunotherapy for a variety of autoimmune illnesses since it has been shown to interfere with T-cell signaling and stimulation [138]. A clinical study using Abatacept for the treatment of COVID-19 patients was just finished and reported beneficial effects in reducing the inflammatory response.

Recently a clinical study employing the combination of Abatacept and the COVID-19 vaccine was conducted by the University of Alabama. Abatacept may reduce the persistence of viral infection and result in milder symptoms, according to an epidemiological study [161]. This implies that the use of CTLA-4-based therapy can be an effective approach to managing patients with COVID-19. TGF-beta, an immunoregulatory molecule generated from Tregs, is also thought to be a target for SARS-CoV-2 therapy in addition to CTLA-4. According to Vaz de Paula et al. (2021), TGF-beta may have a role in both the fluid balance of the lung and the development of lung fibrosis [162]. In order to prevent the establishment of inflammation in the lungs, inhibiting TGF-beta by neutralizing and eliminating TGF-beta using antibodies and/or TGF-beta inhibitors becomes a potential strategy [163], and this can be employed in the management of COVID-19 patients with severe lung damage [164].

A range of studies consisting of COVID-19 patients showed that Notch4 expression was elevated on Tregs and correlated with illness severity, death, and healing. In viral respiratory illnesses, such as SARS-CoV-2 and influenza, the Notch4-amphiregulin nexus has been discovered as a presumed target of treatment [165,166]. In order to stabilize FOXP3 expression and further control the development and function of Tregs, the pathway of Notch4 and Notch ligand delta-like ligand 4 (DLL4) is discovered to elevate H3K4me3 around the *foxp3* locus [167]. The above information shows that disturbing or interfering along the Notch4-DLL4 axis can be a potential therapeutic approach to treat COVID-19. It is also important to remember the multiple functions of FOXP3+ Tregs during viral infection [168,169]. Prior to Treg-based treatment, it is important to thoroughly understand the continuous fluctuations of Tregs, especially their percentage, suppressive function, and FOXP3 stability during various phases of COVID-19 [30,170].

## 7. Conclusions and Future Perspectives

It has been shown that virus-specific T-cell responses, particularly those of regulatory T cells (Tregs), affect tissue damage in respiratory illnesses. Tregs have been shown to play an important role in the pathogenesis of COVID-19 because of their wide range of immunosuppressive activities. Tregs inhibit not only the cells of the innate immune response, such as dendritic cells, natural killer cells, and macrophages, but also the cells of the adaptive immune response, including B and T cells. One of the most important aspects of Tregs in COVID-19 is their capability to limit the excessive release of cytokines which is the major reason for mortality and morbidity in COVID-19 patients. Increases in Tregs have been discovered to be a critical feature during the early stages of SARS-CoV-2 infection because they may reduce CD8+ T cells’ ability to mount an effective antiviral immune response, which can have a negative impact on COVID-19 disease prognosis. Nevertheless, Tregs have been found to be decreased or non-functional in severely infected patients with COVID-19. According to many studies, Tregs were elevated in COVID-19 patients and were detrimental to the disease’s development. Contradictorily, cytokine storm or exaggerated immune response has been linked to lower Treg levels, which leads to a poor prognosis of the disease. It is currently unclear how the proportion of regulatory T cells in COVID-19 changed, particularly when taking into account the biphasic roles of Tregs during the course of the SARS-CoV-2 infection. Tregs are helpful in suppressing inflammation as the illness advances. Hence, to re-establish antiviral immune responses, strategies that target Tregs and lessen their suppressive activity may be helpful, particularly in elderly individuals with immuno-compromised immunity. It is essential to make use of methods that either induce or enlarge Tregs in these individuals in order to bring down the level of hyperinflammation and tissue damage.

Severely infected individuals with COVID-19 have also been shown to have alterations in the phenotypic traits of their Tregs, as well as elevated FoxP3 expression and a distinct transcriptional pattern that is strikingly similar to that of tumor Tregs. In severely infected patients with COVID-19, typical cell growth and enhanced effector functions, particularly ENTPD1, LAG3, and LRRC32, have been noted. Scientists are still figuring out the precise relevance of such perturbations in the Tregs of severely infected patients. Additionally, why such perturbations arise is yet to be resolved. Interestingly, the roles of Tregs have been studied in patients with long COVID. Recent analysis has revealed that Treg dysregulations/perturbations persist in long COVID patients even years after their original SARS-CoV-2 infection. However, the exact roles are still not clear. However, there is no doubt that several studies reported the positive results of several Tregs-based therapeutic interventions. The effectiveness of the adoptive transfer of Tregs has been reported to alleviate the disease severity. Additionally, a low dose of IL-2 has been found to be effective in the generation and stimulation of Tregs in severely infected COVID-19 patients. Many studies have reported the potential uses of CAR-Treg in the treatment of SARS-CoV-2. Furthermore, many small molecules have been found to induce Tregs, which can alleviate the cytokine storm. While many studies have shown the therapeutic potentialities of Tregs-mediated therapies, it is essential to study the effectiveness of Tregs in detail. Tregs should be investigated further as possible treatment targets and prognostic indicators in COVID-19. To draw firmer findings, more research is necessary. This research should include more patients with moderate and severe disorders as well as proven techniques for Treg characterization and Tregs’ concentration measurement. Likewise, studies on individuals who received various COVID-19 immunizations are desperately required to ascertain if Tregs in blood fluctuate after vaccinations and whether these alterations may link to the beneficial effects of immunizations.

## Data Availability

All data are available in this manuscript.

## Abbreviations

ACE2	angiotensin converting enzyme 2
APCs	antigen-presenting cells
BCR	B cell receptor
BALF	bronchoalveolar lavage fluid
COVID-19	coronavirus disease 19
CTLA-4	cytotoxic T lymphocyte associated antigen-4
FoxP3	Forkhead box P3
LAG-3	lymphocyte activation gene 3
MHC	major histocompatibility complex
MHC II	major histocompatibility complex class II
mAbs	monoclonal antibodies
nAbs	neutralizing antibodies
pTregs	peripheral Tregs
PD-L1	programmed death -ligand 1
SARS-CoV-2	severe acute respiratory syndrome coronavirus 2
Tregs	T regulatory cells
tTregs	thymic Tregs
TGF-beta	transforming growth factor-beta 1
TLRs	Toll like receptors
Th1	T helper 1
Th2	T helper 2
Th17	T helper 17
TCR	T cell receptor

## References

[1] Alahdal M., Elkord E. (2022). Exhaustion and Over-Activation of Immune Cells in COVID-19: Challenges and Therapeutic Opportunities. Clin. Immunol..

[2] Dhawan M., Saied A.A., Mitra S., Alhumaydhi F.A., Emran T.B., Wilairatana P. (2022). Omicron Variant (B.1.1.529) and Its Sublineages: What Do We Know so Far amid the Emergence of Recombinant Variants of SARS-CoV-2?. Biomed. Pharmacother..

[3] Ahmad S., Hatmal M.M., Lambuk L., Al-Hatamleh M.A.I., Alshaer W., Mohamud R. (2021). The Role of TNFR2^+^ Tregs in COVID-19: An Overview and a Potential Therapeutic Strategy. Life Sci..

[4] Ahmed J.Q., Maulud S.Q., Dhawan M., Priyanka, Choudhary O.P., Jalal P.J., Ali R.K., Tayib G.A., Hasan D.A. (2022). MicroRNAs in the Development of Potential Therapeutic Targets against COVID-19: A Narrative Review. J. Infect. Public Health.

[5] Tandel N., Negi S., Dalai S.K., Tyagi R.K. (2023). Role of natural killer and B cell interaction in inducing pathogen specific immune responses. Int. Rev. Immunol..

[6] Kudlay D., Kofiadi I., Khaitov M. (2022). Peculiarities of the T Cell Immune Response in COVID-19. Vaccines.

[7] Moss P. (2022). The T Cell Immune Response against SARS-CoV-2. Nat. Immunol..

[8] Chavda V.P., Mishra T., Vuppu S. (2022). Immunological Studies to Understand Hybrid/Recombinant Variants of SARS-CoV-2. Vaccines.

[9] Arish M., Qian W., Narasimhan H., Sun J. (2022). COVID-19 Immunopathology: From Acute Diseases to Chronic Sequelae. J. Med. Virol..

[10] Liu J., Chandrashekar A., Sellers D., Barrett J., Jacob-Dolan C., Lifton M., McMahan K., Sciacca M., VanWyk H., Wu C. (2022). Vaccines Elicit Highly Conserved Cellular Immunity to SARS-CoV-2 Omicron. Nature.

[11] Su Y., Chen D., Yuan D., Lausted C., Choi J., Dai C.L., Voillet V., Duvvuri V.R., Scherler K., Troisch P. (2020). Multi-Omics Resolves a Sharp Disease-State Shift between Mild and Moderate COVID-19. Cell.

[12] Mathew D., Giles J.R., Baxter A.E., Oldridge D.A., Greenplate A.R., Wu J.E., Alanio C., Kuri-Cervantes L., Pampena M.B., D’Andrea K. (2020). Deep Immune Profiling of COVID-19 Patients Reveals Distinct Immunotypes with Therapeutic Implications. Science.

[13] Dhawan M., Rabaan A.A., Fawarah M.M.A., Almuthree S.A., Alsubki R.A., Alfaraj A.H., Mashraqi M.M., Alshamrani S.A., Abduljabbar W.A., Alwashmi A.S.S. (2023). Updated Insights into the T Cell-Mediated Immune Response against SARS-CoV-2: A Step towards Efficient and Reliable Vaccines. Vaccines.

[14] Le Bert N., Clapham H.E., Tan A.T., Chia W.N., Tham C.Y.L., Lim J.M., Kunasegaran K., Tan L.W.L., Dutertre C.-A., Shankar N. (2021). Highly Functional Virus-Specific Cellular Immune Response in Asymptomatic SARS-CoV-2 Infection. J. Exp. Med..

[15] Grau-Expósito J., Sánchez-Gaona N., Massana N., Suppi M., Astorga-Gamaza A., Perea D., Rosado J., Falcó A., Kirkegaard C., Torrella A. (2021). Peripheral and Lung Resident Memory T Cell Responses against SARS-CoV-2. Nat. Commun..

[16] Goswami T.K., Singh M., Dhawan M., Mitra S., Emran T.B., Rabaan A.A., Mutair A.A., Alawi Z.A., Alhumaid S., Dhama K. (2022). Regulatory T Cells (Tregs) and Their Therapeutic Potential against Autoimmune Disorders—Advances and Challenges. Hum. Vaccines Immunother..

[17] Gao M., Liu Y., Guo M., Wang Q., Wang Y., Fan J., Shen Y., Hou J., Wan Y., Zhu Z. (2020). Regulatory CD4^+^ and CD8^+^ T Cells Are Negatively Correlated with CD4^+^/CD8^+^ T Cell Ratios in Patients Acutely Infected with SARS-CoV-2. J. Leukoc. Biol..

[18] Hillaire M., Rimmelzwaan G., Kreijtz J. (2013). Clearance of Influenza Virus Infections by T Cells: Risk of Collateral Damage?. Curr. Opin. Virol..

[19] Wang H., Wang Z., Cao W., Wu Q., Yuan Y., Zhang X. (2021). Regulatory T Cells in COVID-19. Aging Dis..

[20] Galván-Peña S., Leon J., Chowdhary K., Michelson D.A., Vijaykumar B., Yang L., Magnuson A.M., Chen F., Manickas-Hill Z., Piechocka-Trocha A. (2021). Profound Treg Perturbations Correlate with COVID-19 Severity. Proc. Natl. Acad. Sci. USA.

[21] Wang Y., Zheng J., Islam M.S., Yang Y., Hu Y., Chen X. (2021). The Role of CD4^+^FoxP3^+^ Regulatory T Cells in the Immunopathogenesis of COVID-19: Implications for Treatment. Int. J. Biol. Sci..

[22] Seepathomnarong P., Ongarj J., Sophonmanee R., Seeyankem B., Chusri S., Surasombatpattana S., Pinpathomrat N. (2022). Regulatory T Cells Decreased during Recovery from Mild COVID-19. Viruses.

[23] Haunhorst S., Bloch W., Javelle F., Krüger K., Baumgart S., Drube S., Lemhöfer C., Reuken P., Stallmach A., Müller M. (2022). A Scoping Review of Regulatory T Cell Dynamics in Convalescent COVID-19 Patients—Indications for Their Potential Involvement in the Development of Long COVID?. Front. Immunol..

[24] Baecher-Allan C., Brown J.A., Freeman G.J., Hafler D.A. (2001). CD4^+^CD25high Regulatory Cells in Human Peripheral Blood. J. Immunol..

[25] Maekawa D., Riblet S.M., Whang P., Hurley D.J., Garcia M. (2021). Activation of Cytotoxic Lymphocytes and Presence of Regulatory T Cells in the Trachea of Non-Vaccinated and Vaccinated Chickens as a Recall to an Infectious Laryngotracheitis Virus (ILTV) Challenge. Vaccines.

[26] Panduro M., Benoist C., Mathis D. (2016). Tissue Tregs. Annu. Rev. Immunol..

[27] McRitchie B.R., Akkaya B. (2022). Exhaust the Exhausters: Targeting Regulatory T Cells in the Tumor Microenvironment. Front. Immunol..

[28] Magnuson A.M., Kiner E., Ergun A., Park J.S., Asinovski N., Ortiz-Lopez A., Kilcoyne A., Paoluzzi-Tomada E., Weissleder R., Mathis D. (2018). Identification and Validation of a Tumor-Infiltrating Treg Transcriptional Signature Conserved across Species and Tumor Types. Proc. Natl. Acad. Sci. USA.

[29] Lund J.M., Hsing L., Pham T.T., Rudensky A.Y. (2008). Coordination of Early Protective Immunity to Viral Infection by Regulatory T Cells. Science.

[30] Xu Z., Jiang X., Dai X., Li B. (2022). The Dynamic Role of FOXP3^+^ Tregs and Their Potential Therapeutic Applications During SARS-CoV-2 Infection. Front. Immunol..

[31] Sakaguchi S., Sakaguchi N., Asano M., Itoh M., Toda M. (1995). Immunologic Self-Tolerance Maintained by Activated T Cells Expressing IL-2 Receptor Alpha-Chains (CD25). Breakdown of a Single Mechanism of Self-Tolerance Causes Various Autoimmune Diseases. J. Immunol..

[32] Zenclussen A.C., Gerlof K., Zenclussen M.L., Ritschel S., Zambon Bertoja A., Fest S., Hontsu S., Ueha S., Matsushima K., Leber J. (2006). Regulatory T Cells Induce a Privileged Tolerant Microenvironment at the Fetal-Maternal Interface. Eur. J. Immunol..

[33] Palomares O., Rückert B., Jartti T., Kücüksezer U.C., Puhakka T., Gomez E., Fahrner H.B., Speiser A., Jung A., Kwok W.W. (2012). Induction and Maintenance of Allergen-Specific FOXP3^+^ Treg Cells in Human Tonsils as Potential First-Line Organs of Oral Tolerance. J. Allergy Clin. Immunol..

[34] Di Ianni M., Falzetti F., Carotti A., Terenzi A., Castellino F., Bonifacio E., Del Papa B., Zei T., Ostini R.I., Cecchini D. (2011). Tregs Prevent GVHD and Promote Immune Reconstitution in HLA-Haploidentical Transplantation. Blood.

[35] Traxinger B.R., Richert-Spuhler L.E., Lund J.M. (2022). Mucosal Tissue Regulatory T Cells Are Integral in Balancing Immunity and Tolerance at Portals of Antigen Entry. Mucosal Immunol..

[36] Shevach E.M., Thornton A.M. (2014). tTregs, pTregs, and iTregs: Similarities and Differences. Immunol. Rev..

[37] Liu W., Putnam A.L., Xu-yu Z., Szot G.L., Lee M.R., Zhu S., Gottlieb P.A., Kapranov P., Gingeras T.R., de St. Groth B.F. (2006). CD127 Expression Inversely Correlates with FoxP3 and Suppressive Function of Human CD4^+^ T Reg Cells. J. Exp. Med..

[38] Tyagi R.K., Jacobse J., Li J., Allaman M.M., Otipoby K.L., Sampson E.R., Wilson K.T., Goettel J.A. (2021). HLA-Restriction of Human Treg Cells Is Not Required for Therapeutic Efficacy of Low-Dose IL-2 in Humanized Mice. Front. Immunol..

[39] Akkaya B., Shevach E.M. (2020). Regulatory T Cells: Master Thieves of the Immune System. Cell. Immunol..

[40] Shevach E.M. (2009). Mechanisms of Foxp3^+^ T Regulatory Cell-Mediated Suppression. Immunity.

[41] Cao X., Cai S.F., Fehniger T.A., Song J., Collins L.I., Piwnica-Worms D.R., Ley T.J. (2007). Granzyme B and Perforin Are Important for Regulatory T Cell-Mediated Suppression of Tumor Clearance. Immunity.

[42] Borsellino G., Kleinewietfeld M., Di Mitri D., Sternjak A., Diamantini A., Giometto R., Höpner S., Centonze D., Bernardi G., Dell’Acqua M.L. (2007). Expression of Ectonucleotidase CD39 by Foxp3^+^ Treg Cells: Hydrolysis of Extracellular ATP and Immune Suppression. Blood.

[43] Deaglio S., Dwyer K.M., Gao W., Friedman D., Usheva A., Erat A., Chen J.-F., Enjyoji K., Linden J., Oukka M. (2007). Adenosine Generation Catalyzed by CD39 and CD73 Expressed on Regulatory T Cells Mediates Immune Suppression. J. Exp. Med..

[44] Tyagi R.K., Miles B., Parmar R., Garg N.K., Dalai S.K., Baban B., Cutler C.W. (2017). Human IDO-competent, long-lived immunoregulatory dendritic cells induced by intracellular pathogen, and their fate in humanized mice. Sci. Rep..

[45] Zarek P.E., Huang C.-T., Lutz E.R., Kowalski J., Horton M.R., Linden J., Drake C.G., Powell J.D. (2008). A2A Receptor Signaling Promotes Peripheral Tolerance by Inducing T-Cell Anergy and the Generation of Adaptive Regulatory T Cells. Blood.

[46] Qureshi O.S., Zheng Y., Nakamura K., Attridge K., Manzotti C., Schmidt E.M., Baker J., Jeffery L.E., Kaur S., Briggs Z. (2011). Trans-Endocytosis of CD80 and CD86: A Molecular Basis for the Cell-Extrinsic Function of CTLA-4. Science.

[47] Fallarino F., Grohmann U., Hwang K.W., Orabona C., Vacca C., Bianchi R., Belladonna M.L., Fioretti M.C., Alegre M.-L., Puccetti P. (2003). Modulation of Tryptophan Catabolism by Regulatory T Cells. Nat. Immunol..

[48] Yan Z., Garg S.K., Banerjee R. (2010). Regulatory T Cells Interfere with Glutathione Metabolism in Dendritic Cells and T Cells. J. Biol. Chem..

[49] Liang B., Workman C., Lee J., Chew C., Dale B.M., Colonna L., Flores M., Li N., Schweighoffer E., Greenberg S. (2008). Regulatory T Cells Inhibit Dendritic Cells by Lymphocyte Activation Gene-3 Engagement of MHC Class II. J. Immunol..

[50] Gotot J., Gottschalk C., Leopold S., Knolle P.A., Yagita H., Kurts C., Ludwig-Portugall I. (2012). Regulatory T Cells Use Programmed Death 1 Ligands to Directly Suppress Autoreactive B Cells in Vivo. Proc. Natl. Acad. Sci. USA.

[51] Samaan E., Elmaria M.O., Khedr D., Gaber T., Elsayed A.G., Shenouda R.N., Gamal H., Shahin D., Abousamra N.K., Shemies R. (2022). Characterization of Regulatory T Cells in SARS-CoV-2 Infected Hemodialysis Patients: Relation to Clinical and Radiological Severity. BMC Nephrol..

[52] Vick S.C., Frutoso M., Mair F., Konecny A.J., Greene E., Wolf C.R., Logue J.K., Franko N.M., Boonyaratanakornkit J., Gottardo R. (2021). A Regulatory T Cell Signature Distinguishes the Immune Landscape of COVID-19 Patients from Those with Other Respiratory Infections. Sci. Adv..

[53] Ronit A., Berg R.M.G., Bay J.T., Haugaard A.K., Ahlström M.G., Burgdorf K.S., Ullum H., Rørvig S.B., Tjelle K., Foss N.B. (2021). Compartmental Immunophenotyping in COVID-19 ARDS: A Case Series. J. Allergy Clin. Immunol..

[54] Yang J., Zhong M., Hong K., Yang Q., Zhang E., Zhou D., Xia J., Chen Y., Sun M., Zhao B. (2021). Characteristics of T-cell Responses in COVID-19 Patients with Prolonged SARS-CoV-2 Positivity—A Cohort Study. Clin. Transl. Immunol..

[55] De Biasi S., Meschiari M., Gibellini L., Bellinazzi C., Borella R., Fidanza L., Gozzi L., Iannone A., Lo Tartaro D., Mattioli M. (2020). Marked T Cell Activation, Senescence, Exhaustion and Skewing towards TH17 in Patients with COVID-19 Pneumonia. Nat. Commun..

[56] Zheng H., Li H., Guo L., Liang Y., Li J., Wang X., Hu Y., Wang L., Liao Y., Yang F. (2020). Virulence and Pathogenesis of SARS-CoV-2 Infection in Rhesus Macaques: A Nonhuman Primate Model of COVID-19 Progression. PLOS Pathog..

[57] Rajamanickam A., Pavan Kumar N., Pandiaraj A.N., Selvaraj N., Munisankar S., Renji R.M., Venkataramani V., Murhekar M., Thangaraj J.W.V., Muthusamy S.K. (2022). Characterization of memory T cell subsets and common γ-chain cytokines in convalescent COVID-19 individuals. J. Leukoc. Biol..

[58] Mahmoud Salehi Khesht A., Karpisheh V., Qubais Saeed B., Olegovna Zekiy A., Yapanto L.M., Nabi Afjadi M., Aksoun M., Nasr Esfahani M., Aghakhani F., Movahed M. (2021). Different T Cell Related Immunological Profiles in COVID-19 Patients Compared to Healthy Controls. Int. Immunopharmacol..

[59] Sadeghi A., Tahmasebi S., Mahmood A., Kuznetsova M., Valizadeh H., Taghizadieh A., Nazemiyeh M., Aghebati-Maleki L., Jadidi-Niaragh F., Abbaspour-Aghdam S. (2020). Th17 and Treg Cells Function in SARS-CoV2 Patients Compared with Healthy Controls. J. Cell. Physiol..

[60] Meckiff B.J., Ramírez-Suástegui C., Fajardo V., Chee S.J., Kusnadi A., Simon H., Eschweiler S., Grifoni A., Pelosi E., Weiskopf D. (2020). Imbalance of Regulatory and Cytotoxic SARS-CoV-2-Reactive CD4^+^ T Cells in COVID-19. Cell.

[61] Caldrer S., Mazzi C., Bernardi M., Prato M., Ronzoni N., Rodari P., Angheben A., Piubelli C., Tiberti N. (2021). Regulatory T Cells as Predictors of Clinical Course in Hospitalised COVID-19 Patients. Front. Immunol..

[62] Qin C., Zhou L., Hu Z., Zhang S., Yang S., Tao Y., Xie C., Ma K., Shang K., Wang W. (2020). Dysregulation of Immune Response in Patients With Coronavirus 2019 (COVID-19) in Wuhan, China. Clin. Infect. Dis..

[63] Wang W., Su B., Pang L., Qiao L., Feng Y., Ouyang Y., Guo X., Shi H., Wei F., Su X. (2020). High-Dimensional Immune Profiling by Mass Cytometry Revealed Immunosuppression and Dysfunction of Immunity in COVID-19 Patients. Cell. Mol. Immunol..

[64] Jia R., Wang X., Liu P., Liang X., Ge Y., Tian H., Chang H., Zhou H., Zeng M., Xu J. (2020). Mild Cytokine Elevation, Moderate CD4^+^ T Cell Response and Abundant Antibody Production in Children with COVID-19. Virol. Sin..

[65] Szabo P.A., Dogra P., Gray J.I., Wells S.B., Connors T.J., Weisberg S.P., Krupska I., Matsumoto R., Poon M.M.L., Idzikowski E. (2021). Longitudinal Profiling of Respiratory and Systemic Immune Responses Reveals Myeloid Cell-Driven Lung Inflammation in Severe COVID-19. Immunity.

[66] Feng Y., Arvey A., Chinen T., van der Veeken J., Gasteiger G., Rudensky A.Y. (2014). Control of the Inheritance of Regulatory T Cell Identity by a Cis Element in the Foxp3 Locus. Cell.

[67] Yang X.O., Nurieva R., Martinez G.J., Kang H.S., Chung Y., Pappu B.P., Shah B., Chang S.H., Schluns K.S., Watowich S.S. (2008). Molecular Antagonism and Plasticity of Regulatory and Inflammatory T Cell Programs. Immunity.

[68] Simsek A., Kizmaz M.A., Cagan E., Dombaz F., Tezcan G., Asan A., Demir H.I., Bal S.H., Ermis D.Y., Dilektaslı A.G. (2022). Assessment of CD39 Expression in Regulatory T-cell Subsets by Disease Severity in Adult and Juvenile COVID-19 Cases. J. Med. Virol..

[69] Ahern D.J., Ai Z., Ainsworth M., Allan C., Allcock A., Angus B., Ansari M.A., Arancibia-Cárcamo C.V., Aschenbrenner D., Attar M. (2022). A Blood Atlas of COVID-19 Defines Hallmarks of Disease Severity and Specificity. Cell.

[70] Chen G., Wu D., Guo W., Cao Y., Huang D., Wang H., Wang T., Zhang X., Chen H., Yu H. (2020). Clinical and Immunological Features of Severe and Moderate Coronavirus Disease 2019. J. Clin. Investig..

[71] Tan L., Wang Q., Zhang D., Ding J., Huang Q., Tang Y.-Q., Wang Q., Miao H. (2020). Lymphopenia Predicts Disease Severity of COVID-19: A Descriptive and Predictive Study. Signal Transduct. Target. Ther..

[72] Ren X., Wen W., Fan X., Hou W., Su B., Cai P., Li J., Liu Y., Tang F., Zhang F. (2021). COVID-19 Immune Features Revealed by a Large-Scale Single-Cell Transcriptome Atlas. Cell.

[73] Shen X.-R., Geng R., Li Q., Chen Y., Li S.-F., Wang Q., Min J., Yang Y., Li B., Jiang R.-D. (2022). ACE2-Independent Infection of T Lymphocytes by SARS-CoV-2. Signal Transduct. Target. Ther..

[74] Kratzer B., Trapin D., Ettel P., Körmöczi U., Rottal A., Tuppy F., Feichter M., Gattinger P., Borochova K., Dorofeeva Y. (2020). Immunological Imprint of COVID-19 on Human Peripheral Blood Leukocyte Populations. Allergy.

[75] Mohebbi S.R., Baghaei K., Rostami-Nejad M., Mojarad E.N., Mirjalali H., Yadegar A., Asri N., Abdoulahi S., Assadzadeh Aghdaei H. (2020). Significant Changes of CD4, FOXP3, CD25, and IL6 Expression Level in Iranian COVID-19 Patients. Gastroenterol. Hepatol. Bed Bench.

[76] Kalfaoglu B., Almeida-Santos J., Tye C.A., Satou Y., Ono M. (2020). T-Cell Hyperactivation and Paralysis in Severe COVID-19 Infection Revealed by Single-Cell Analysis. Front. Immunol..

[77] Thibaudin M., Fumet J.-D., Bon M., Hampe L., Limagne E., Ghiringhelli F. (2020). Immunological Features of Coronavirus Disease 2019 in Patients with Cancer. Eur. J. Cancer.

[78] Gupta S., Su H., Narsai T., Agrawal S. (2021). SARS-CoV-2-Associated T-Cell Responses in the Presence of Humoral Immunodeficiency. Int. Arch. Allergy Immunol..

[79] Corthay A. (2009). How Do Regulatory T Cells Work?. Scand. J. Immunol..

[80] Dejaco C., Duftner C., Grubeck-Loebenstein B., Schirmer M. (2006). Imbalance of Regulatory T Cells in Human Autoimmune Diseases. Immunology.

[81] Komatsu N., Okamoto K., Sawa S., Nakashima T., Oh-hora M., Kodama T., Tanaka S., Bluestone J.A., Takayanagi H. (2013). Pathogenic Conversion of Foxp3^+^ T Cells into TH17 Cells in Autoimmune Arthritis. Nat. Med..

[82] Jasim S.A., Mahdi R.S., Bokov D.O., Najm M.A.A., Sobirova G.N., Bafoyeva Z.O., Taifi A., Alkadir O.K.A., Mustafa Y.F., Mirzaei R. (2022). The Deciphering of the Immune Cells and Marker Signature in COVID-19 Pathogenesis: An Update. J. Med. Virol..

[83] Brown B., Ojha V., Fricke I., Al-Sheboul S.A., Imarogbe C., Gravier T., Green M., Peterson L., Koutsaroff I.P., Demir A. (2023). Innate and Adaptive Immunity during SARS-CoV-2 Infection: Biomolecular Cellular Markers and Mechanisms. Vaccines.

[84] Neumann J., Prezzemolo T., Vanderbeke L., Roca C.P., Gerbaux M., Janssens S., Willemsen M., Burton O., Van Mol P., Van Herck Y. (2020). Increased IL-10-producing Regulatory T Cells Are Characteristic of Severe Cases of COVID-19. Clin. Transl. Immunol..

[85] Filbin M.R., Mehta A., Schneider A.M., Kays K.R., Guess J.R., Gentili M., Fenyves B.G., Charland N.C., Gonye A.L.K., Gushterova I. (2021). Longitudinal Proteomic Analysis of Severe COVID-19 Reveals Survival-Associated Signatures, Tissue-Specific Cell Death, and Cell-Cell Interactions. Cell Rep. Med..

[86] LIU M., GUO S., STILES J.K. (2011). The Emerging Role of CXCL10 in Cancer (Review). Oncol. Lett..

[87] Veiga-Parga T., Sehrawat S., Rouse B.T. (2013). Role of Regulatory T Cells during Virus Infection. Immunol. Rev..

[88] Sehrawat S., Rouse B.T. (2011). Tregs and Infections: On the Potential Value of Modifying Their Function. J. Leukoc. Biol..

[89] Bettelli E., Carrier Y., Gao W., Korn T., Strom T.B., Oukka M., Weiner H.L., Kuchroo V.K. (2006). Reciprocal Developmental Pathways for the Generation of Pathogenic Effector TH17 and Regulatory T Cells. Nature.

[90] Sefik E., Geva-Zatorsky N., Oh S., Konnikova L., Zemmour D., McGuire A.M., Burzyn D., Ortiz-Lopez A., Lobera M., Yang J. (2015). Individual Intestinal Symbionts Induce a Distinct Population of RORγ^+^ Regulatory T Cells. Science.

[91] Ohnmacht C., Park J.-H., Cording S., Wing J.B., Atarashi K., Obata Y., Gaboriau-Routhiau V., Marques R., Dulauroy S., Fedoseeva M. (2015). The Microbiota Regulates Type 2 Immunity through RORγt^+^ T Cells. Science.

[92] Hagenstein J., Melderis S., Nosko A., Warkotsch M.T., Richter J.V., Ramcke T., Herrnstadt G.R., Scheller J., Yan I., Mittrücker H.-W. (2019). A Novel Role for IL-6 Receptor Classic Signaling: Induction of RORγt^+^Foxp3^+^ Tregs with Enhanced Suppressive Capacity. J. Am. Soc. Nephrol..

[93] Fujimoto M., Nakano M., Terabe F., Kawahata H., Ohkawara T., Han Y., Ripley B., Serada S., Nishikawa T., Kimura A. (2011). The Influence of Excessive IL-6 Production In Vivo on the Development and Function of Foxp3^+^ Regulatory T Cells. J. Immunol..

[94] Mijnheer G., Lutter L., Mokry M., van der Wal M., Scholman R., Fleskens V., Pandit A., Tao W., Wekking M., Vervoort S. (2021). Conserved Human Effector Treg Cell Transcriptomic and Epigenetic Signature in Arthritic Joint Inflammation. Nat. Commun..

[95] Arpaia N., Green J.A., Moltedo B., Arvey A., Hemmers S., Yuan S., Treuting P.M., Rudensky A.Y. (2015). A Distinct Function of Regulatory T Cells in Tissue Protection. Cell.

[96] Harrison O.J., Srinivasan N., Pott J., Schiering C., Krausgruber T., Ilott N.E., Maloy K.J. (2015). Epithelial-Derived IL-18 Regulates Th17 Cell Differentiation and Foxp3^+^ Treg Cell Function in the Intestine. Mucosal Immunol..

[97] Harb H., Benamar M., Lai P.S., Contini P., Griffith J.W., Crestani E., Schmitz-Abe K., Chen Q., Fong J., Marri L. (2021). Notch4 Signaling Limits Regulatory T-Cell-Mediated Tissue Repair and Promotes Severe Lung Inflammation in Viral Infections. Immunity.

[98] Peligero-Cruz C., Givony T., Sebé-Pedrós A., Dobeš J., Kadouri N., Nevo S., Roncato F., Alon R., Goldfarb Y., Abramson J. (2020). IL18 Signaling Promotes Homing of Mature Tregs into the Thymus. ELife.

[99] Buckley P.R., Lee C.H., Pereira Pinho M., Ottakandathil Babu R., Woo J., Antanaviciute A., Simmons A., Ogg G., Koohy H. (2022). HLA-dependent Variation in SARS-CoV-2 CD8^+^ T Cell Cross-reactivity with Human Coronaviruses. Immunology.

[100] Overacre-Delgoffe A.E., Chikina M., Dadey R.E., Yano H., Brunazzi E.A., Shayan G., Horne W., Moskovitz J.M., Kolls J.K., Sander C. (2017). Interferon-γ Drives Treg Fragility to Promote Anti-Tumor Immunity. Cell.

[101] Yang J., Zhang E., Zhong M., Yang Q., Hong K., Shu T., Zhou D., Xiang J., Xia J., Zhou X. (2020). Longitudinal Characteristics of T Cell Responses in Asymptomatic SARS-CoV-2 Infection. Virol. Sin..

[102] Benamar M., Chen Q., Chou J., Julé A.M., Boudra R., Contini P., Crestani E., Lai P.S., Wang M., Fong J. (2023). The Notch1/CD22 Signaling Axis Disrupts Treg Function in SARS-CoV-2–Associated Multisystem Inflammatory Syndrome in Children. J. Clin. Investig..

[103] Guan W., Ni Z., Hu Y., Liang W., Ou C., He J., Liu L., Shan H., Lei C., Hui D.S.C. (2020). Clinical Characteristics of Coronavirus Disease 2019 in China. N. Engl. J. Med..

[104] Gupta A., Madhavan M.V., Sehgal K., Nair N., Mahajan S., Sehrawat T.S., Bikdeli B., Ahluwalia N., Ausiello J.C., Wan E.Y. (2020). Extrapulmonary Manifestations of COVID-19. Nat. Med..

[105] Ackermann M., Verleden S.E., Kuehnel M., Haverich A., Welte T., Laenger F., Vanstapel A., Werlein C., Stark H., Tzankov A. (2020). Pulmonary Vascular Endothelialitis, Thrombosis, and Angiogenesis in COVID-19. N. Engl. J. Med..

[106] Henderson L.A., Canna S.W., Schulert G.S., Volpi S., Lee P.Y., Kernan K.F., Caricchio R., Mahmud S., Hazen M.M., Halyabar O. (2020). On the Alert for Cytokine Storm: Immunopathology in COVID-19. Arthritis Rheumatol..

[107] Ballering A.V., van Zon S.K.R., olde Hartman T.C., Rosmalen J.G.M. (2022). Persistence of Somatic Symptoms after COVID-19 in the Netherlands: An Observational Cohort Study. Lancet.

[108] Soriano J.B., Murthy S., Marshall J.C., Relan P., Diaz J.V. (2022). A Clinical Case Definition of Post-COVID-19 Condition by a Delphi Consensus. Lancet Infect. Dis..

[109] Iqbal F.M., Lam K., Sounderajah V., Clarke J.M., Ashrafian H., Darzi A. (2021). Characteristics and Predictors of Acute and Chronic Post-COVID Syndrome: A Systematic Review and Meta-Analysis. eClinicalMedicine.

[110] Davis H.E., Assaf G.S., McCorkell L., Wei H., Low R.J., Re’em Y., Redfield S., Austin J.P., Akrami A. (2021). Characterizing Long COVID in an International Cohort: 7 Months of Symptoms and Their Impact. eClinicalMedicine.

[111] Giszas B., Trommer S., Schüßler N., Rodewald A., Besteher B., Bleidorn J., Dickmann P., Finke K., Katzer K., Lehmann-Pohl K. (2022). Post-COVID-19 Condition Is Not Only a Question of Persistent Symptoms: Structured Screening Including Health-Related Quality of Life Reveals Two Separate Clusters of Post-COVID. Infection.

[112] Merad M., Blish C.A., Sallusto F., Iwasaki A. (2022). The Immunology and Immunopathology of COVID-19. Science.

[113] Sun B., Tang N., Peluso M.J., Iyer N.S., Torres L., Donatelli J.L., Munter S.E., Nixon C.C., Rutishauser R.L., Rodriguez-Barraquer I. (2021). Characterization and Biomarker Analyses of Post-COVID-19 Complications and Neurological Manifestations. Cells.

[114] Peluso M.J., Lu S., Tang A.F., Durstenfeld M.S., Ho H., Goldberg S.A., Forman C.A., Munter S.E., Hoh R., Tai V. (2021). Markers of Immune Activation and Inflammation in Individuals With Postacute Sequelae of Severe Acute Respiratory Syndrome Coronavirus 2 Infection. J. Infect. Dis..

[115] Galán M., Vigón L., Fuertes D., Murciano-Antón M.A., Casado-Fernández G., Domínguez-Mateos S., Mateos E., Ramos-Martín F., Planelles V., Torres M. (2022). Persistent Overactive Cytotoxic Immune Response in a Spanish Cohort of Individuals With Long-COVID: Identification of Diagnostic Biomarkers. Front. Immunol..

[116] Patterson B.K., Guevara-Coto J., Yogendra R., Francisco E.B., Long E., Pise A., Rodrigues H., Parikh P., Mora J., Mora-Rodríguez R.A. (2021). Immune-Based Prediction of COVID-19 Severity and Chronicity Decoded Using Machine Learning. Front. Immunol..

[117] Utrero-Rico A., Ruiz-Ruigómez M., Laguna-Goya R., Arrieta-Ortubay E., Chivite-Lacaba M., González-Cuadrado C., Lalueza A., Almendro-Vazquez P., Serrano A., Aguado J.M. (2021). A Short Corticosteroid Course Reduces Symptoms and Immunological Alterations Underlying Long-COVID. Biomedicines.

[118] Petrara M.R., Bonfante F., Costenaro P., Cantarutti A., Carmona F., Ruffoni E., Di Chiara C., Zanchetta M., Barzon L., Donà D. (2021). Asymptomatic and Mild SARS-CoV-2 Infections Elicit Lower Immune Activation and Higher Specific Neutralizing Antibodies in Children Than in Adults. Front. Immunol..

[119] Ryan F.J., Hope C.M., Masavuli M.G., Lynn M.A., Mekonnen Z.A., Yeow A.E.L., Garcia-Valtanen P., Al-Delfi Z., Gummow J., Ferguson C. (2022). Long-Term Perturbation of the Peripheral Immune System Months after SARS-CoV-2 Infection. BMC Med..

[120] Wiech M., Chroscicki P., Swatler J., Stepnik D., De Biasi S., Hampel M., Brewinska-Olchowik M., Maliszewska A., Sklinda K., Durlik M. (2022). Remodeling of T Cell Dynamics During Long COVID Is Dependent on Severity of SARS-CoV-2 Infection. Front. Immunol..

[121] Garcia-Gasalla M., Berman-Riu M., Pons J., Rodríguez A., Iglesias A., Martínez-Pomar N., Llompart-Alabern I., Riera M., Ferré Beltrán A., Figueras-Castilla A. (2022). Hyperinflammatory State and Low T1 Adaptive Immune Response in Severe and Critical Acute COVID-19 Patients. Front. Med..

[122] Suvvari T.K., Kutikuppala L.V.S., Tsagkaris C., Corriero A.C., Kandi V. (2021). Post-COVID-19 Complications: Multisystemic Approach. J. Med. Virol..

[123] Ståhlberg M., Reistam U., Fedorowski A., Villacorta H., Horiuchi Y., Bax J., Pitt B., Matskeplishvili S., Lüscher T.F., Weichert I. (2021). Post-COVID-19 Tachycardia Syndrome: A Distinct Phenotype of Post-Acute COVID-19 Syndrome. Am. J. Med..

[124] Afrin L.B., Weinstock L.B., Molderings G.J. (2020). COVID--19 Hyperinflammation and Post-COVID--19 Illness May Be Rooted in Mast Cell Activation Syndrome. Int. J. Infect. Dis..

[125] Whitaker M., Elliott J., Chadeau-Hyam M., Riley S., Darzi A., Cooke G., Ward H., Elliott P. (2022). Persistent COVID-19 Symptoms in a Community Study of 606,434 People in England. Nat. Commun..

[126] Hefazi M., Bolivar-Wagers S., Blazar B.R. (2021). Regulatory T Cell Therapy of Graft-versus-Host Disease: Advances and Challenges. Int. J. Mol. Sci..

[127] Sun J., Han Z.-B., Liao W., Yang S.G., Yang Z., Yu J., Meng L., Wu R., Han Z.C. (2011). Intrapulmonary Delivery of Human Umbilical Cord Mesenchymal Stem Cells Attenuates Acute Lung Injury by Expanding CD4^+^CD25^+^ Forkhead Boxp3 (FOXP3) ^+^ Regulatory T Cells and Balancing Anti- and Pro-Inflammatory Factors. Cell. Physiol. Biochem..

[128] Cao Y., Xu W., Xiong S. (2013). Adoptive Transfer of Regulatory T Cells Protects against Coxsackievirus B3-Induced Cardiac Fibrosis. PLoS ONE.

[129] Gladstone D.E., Kim B.S., Mooney K., Karaba A.H., D’Alessio F.R. (2020). Regulatory T Cells for Treating Patients With COVID-19 and Acute Respiratory Distress Syndrome: Two Case Reports. Ann. Intern. Med..

[130] Hossein-khannazer N., Shokoohian B., Shpichka A., Aghdaei H.A., Timashev P., Vosough M. (2021). An Update to “Novel Therapeutic Approaches for Treatment of COVID-19”. J. Mol. Med..

[131] Zhang Y., Wang X., Li X., Xi D., Mao R., Wu X., Cheng S., Sun X., Yi C., Ling Z. (2020). Potential Contribution of Increased Soluble IL-2R to Lymphopenia in COVID-19 Patients. Cell. Mol. Immunol..

[132] Rabaan A.A., Al-Ahmed S.H., Muhammad J., Khan A., Sule A.A., Tirupathi R., Mutair A.A., Alhumaid S., Al-Omari A., Dhawan M. (2021). Role of Inflammatory Cytokines in COVID-19 Patients: A Review on Molecular Mechanisms, Immune Functions, Immunopathology and Immunomodulatory Drugs to Counter Cytokine Storm. Vaccines.

[133] Hartemann A., Bensimon G., Payan C.A., Jacqueminet S., Bourron O., Nicolas N., Fonfrede M., Rosenzwajg M., Bernard C., Klatzmann D. (2013). Low-Dose Interleukin 2 in Patients with Type 1 Diabetes: A Phase 1/2 Randomised, Double-Blind, Placebo-Controlled Trial. Lancet Diabetes Endocrinol..

[134] Kennedy-Nasser A.A., Ku S., Castillo-Caro P., Hazrat Y., Wu M.-F., Liu H., Melenhorst J., Barrett A.J., Ito S., Foster A. (2014). Ultra Low-Dose IL-2 for GVHD Prophylaxis after Allogeneic Hematopoietic Stem Cell Transplantation Mediates Expansion of Regulatory T Cells without Diminishing Antiviral and Antileukemic Activity. Clin. Cancer Res..

[135] Zhu M.-E., Wang Q., Zhou S., Wang B., Ke L., He P. (2021). Recombinant Interleukin-2 Stimulates Lymphocyte Recovery in Patients with Severe COVID-19. Exp. Ther. Med..

[136] Hou H., Zhang B., Huang H., Luo Y., Wu S., Tang G., Liu W., Mao L., Mao L., Wang F. (2020). Using IL-2R/Lymphocytes for Predicting the Clinical Progression of Patients with COVID-19. Clin. Exp. Immunol..

[137] Jang H.J., Leem A.Y., Chung K.S., Ahn J.Y., Jung J.Y., Kang Y.A., Park M.S., Kim Y.S., Lee S.H. (2021). Soluble IL-2R Levels Predict in-Hospital Mortality in COVID-19 Patients with Respiratory Failure. J. Clin. Med..

[138] Stephen-Victor E., Das M., Karnam A., Pitard B., Gautier J.-F., Bayry J. (2020). Potential of Regulatory T-Cell-Based Therapies in the Management of Severe COVID-19. Eur. Respir. J..

[139] Trotta E., Bessette P.H., Silveria S.L., Ely L.K., Jude K.M., Le D.T., Holst C.R., Coyle A., Potempa M., Lanier L.L. (2018). A Human Anti-IL-2 Antibody That Potentiates Regulatory T Cells by a Structure-Based Mechanism. Nat. Med..

[140] Webster K.E., Walters S., Kohler R.E., Mrkvan T., Boyman O., Surh C.D., Grey S.T., Sprent J. (2009). In Vivo Expansion of T Reg Cells with IL-2–mAb Complexes: Induction of Resistance to EAE and Long-Term Acceptance of Islet Allografts without Immunosuppression. J. Exp. Med..

[141] Hosseini A., Gharibi T., Mohammadzadeh A., Ebrahimi-kalan A., Jadidi-niaragh F., Babaloo Z., Shanehbandi D., Baghbani E., Baradaran B. (2021). Ruxolitinib Attenuates Experimental Autoimmune Encephalomyelitis (EAE) Development as Animal Models of Multiple Sclerosis (MS). Life Sci..

[142] Uraki R., Imai M., Ito M., Shime H., Odanaka M., Okuda M., Kawaoka Y., Yamazaki S. (2021). Foxp3^+^ CD4^+^ Regulatory T Cells Control Dendritic Cells in Inducing Antigen-Specific Immunity to Emerging SARS-CoV-2 Antigens. PLOS Pathog..

[143] Cheng H., Wang L., Yang B., Li D., Wang X., Liu X., Tian N., Huang Q., Feng R., Wang Z. (2020). Cutting Edge: Inhibition of Glycogen Synthase Kinase 3 Activity Induces the Generation and Enhanced Suppressive Function of Human IL-10^+^ FOXP3^+^–Induced Regulatory T Cells. J. Immunol..

[144] Rudd C.E. (2020). GSK-3 Inhibition as a Therapeutic Approach Against SARs CoV2: Dual Benefit of Inhibiting Viral Replication While Potentiating the Immune Response. Front. Immunol..

[145] Sauer S., Bruno L., Hertweck A., Finlay D., Leleu M., Spivakov M., Knight Z.A., Cobb B.S., Cantrell D., O’Connor E. (2008). T Cell Receptor Signaling Controls Foxp3 Expression via PI3K, Akt, and mTOR. Proc. Natl. Acad. Sci. USA.

[146] Zeiser R., Leveson-Gower D.B., Zambricki E.A., Kambham N., Beilhack A., Loh J., Hou J.-Z., Negrin R.S. (2008). Differential Impact of Mammalian Target of Rapamycin Inhibition on CD4^+^CD25^+^Foxp3^+^ Regulatory T Cells Compared with Conventional CD4^+^ T Cells. Blood.

[147] Battaglia M., Stabilini A., Migliavacca B., Horejs-Hoeck J., Kaupper T., Roncarolo M.-G. (2006). Rapamycin Promotes Expansion of Functional CD4^+^CD25^+^FOXP3^+^ Regulatory T Cells of Both Healthy Subjects and Type 1 Diabetic Patients. J. Immunol..

[148] Bischof E., Siow R.C., Zhavoronkov A., Kaeberlein M. (2021). The Potential of Rapalogs to Enhance Resilience against SARS-CoV-2 Infection and Reduce the Severity of COVID-19. Lancet Healthy Longev..

[149] Lu L., Lan Q., Li Z., Zhou X., Gu J., Li Q., Wang J., Chen M., Liu Y., Shen Y. (2014). Critical Role of All—Trans Retinoic Acid in Stabilizing Human Natural Regulatory T Cells under Inflammatory Conditions. Proc. Natl. Acad. Sci. USA.

[150] Elias K.M., Laurence A., Davidson T.S., Stephens G., Kanno Y., Shevach E.M., O’Shea J.J. (2008). Retinoic Acid Inhibits Th17 Polarization and Enhances FoxP3 Expression through a Stat-3/Stat-5 Independent Signaling Pathway. Blood.

[151] Zhou X., Kong N., Wang J., Fan H., Zou H., Horwitz D., Brand D., Liu Z., Zheng S.G. (2010). Cutting Edge: All-Trans Retinoic Acid Sustains the Stability and Function of Natural Regulatory T Cells in an Inflammatory Milieu. J. Immunol..

[152] Morita T., Miyakawa K., Jeremiah S.S., Yamaoka Y., Sada M., Kuniyoshi T., Yang J., Kimura H., Ryo A. (2021). All-Trans Retinoic Acid Exhibits Antiviral Effect against SARS-CoV-2 by Inhibiting 3CLpro Activity. Viruses.

[153] Eggenhuizen P.J., Ng B.H., Ooi J.D. (2020). Treg Enhancing Therapies to Treat Autoimmune Diseases. Int. J. Mol. Sci..

[154] Tang Q., Henriksen K.J., Bi M., Finger E.B., Szot G., Ye J., Masteller E.L., McDevitt H., Bonyhadi M., Bluestone J.A. (2004). In Vitro–Expanded Antigen-Specific Regulatory T Cells Suppress Autoimmune Diabetes. J. Exp. Med..

[155] Wright G.P., Notley C.A., Xue S.-A., Bendle G.M., Holler A., Schumacher T.N., Ehrenstein M.R., Stauss H.J. (2009). Adoptive Therapy with Redirected Primary Regulatory T Cells Results in Antigen-Specific Suppression of Arthritis. Proc. Natl. Acad. Sci. USA.

[156] Tsang J.Y.-S., Tanriver Y., Jiang S., Xue S.-A., Ratnasothy K., Chen D., Stauss H.J., Bucy R.P., Lombardi G., Lechler R. (2008). Conferring Indirect Allospecificity on CD4^+^CD25^+^ Tregs by TCR Gene Transfer Favors Transplantation Tolerance in Mice. J. Clin. Investig..

[157] Zhang Q., Lu W., Liang C.-L., Chen Y., Liu H., Qiu F., Dai Z. (2018). Chimeric Antigen Receptor (CAR) Treg: A Promising Approach to Inducing Immunological Tolerance. Front. Immunol..

[158] Fransson M., Piras E., Burman J., Nilsson B., Essand M., Lu B., Harris R.A., Magnusson P.U., Brittebo E., Loskog A.S. (2012). CAR/FoxP3-Engineered T Regulatory Cells Target the CNS and Suppress EAE upon Intranasal Delivery. J. Neuroinflammation.

[159] Blat D., Zigmond E., Alteber Z., Waks T., Eshhar Z. (2014). Suppression of Murine Colitis and Its Associated Cancer by Carcinoembryonic Antigen-Specific Regulatory T Cells. Mol. Ther..

[160] Skuljec J., Chmielewski M., Happle C., Habener A., Busse M., Abken H., Hansen G. (2017). Chimeric Antigen Receptor-Redirected Regulatory T Cells Suppress Experimental Allergic Airway Inflammation, a Model of Asthma. Front. Immunol..

[161] Michelena X., Borrell H., López-Corbeto M., López-Lasanta M., Moreno E., Pascual-Pastor M., Erra A., Serrat M., Espartal E., Antón S. (2020). Incidence of COVID-19 in a Cohort of Adult and Paediatric Patients with Rheumatic Diseases Treated with Targeted Biologic and Synthetic Disease-Modifying Anti-Rheumatic Drugs. Semin. Arthritis Rheum..

[162] Vaz de Paula C.B., Nagashima S., Liberalesso V., Collete M., da Silva F.P.G., Oricil A.G.G., Barbosa G.S., da Silva G.V.C., Wiedmer D.B., da Silva Dezidério F. (2021). COVID-19: Immunohistochemical Analysis of TGF-β Signaling Pathways in Pulmonary Fibrosis. Int. J. Mol. Sci..

[163] Chen W. (2020). A Potential Treatment of COVID-19 with TGF-β Blockade. Int. J. Biol. Sci..

[164] He J., Zhang R., Shao M., Zhao X., Miao M., Chen J., Liu J., Zhang X., Zhang X., Jin Y. (2019). Efficacy and Safety of Low-Dose IL-2 in the Treatment of Systemic Lupus Erythematosus: A Randomised, Double-Blind, Placebo-Controlled Trial. Ann. Rheum. Dis..

[165] Chaudhary B., Elkord E. (2016). Regulatory T Cells in the Tumor Microenvironment and Cancer Progression: Role and Therapeutic Targeting. Vaccines.

[166] Gliwiński M., Iwaszkiewicz-Grześ D., Trzonkowski P. (2017). Cell-Based Therapies with T Regulatory Cells. BioDrugs.

[167] Ting H.-A., de Almeida Nagata D., Rasky A.J., Malinczak C.-A., Maillard I.P., Schaller M.A., Lukacs N.W. (2018). Notch Ligand Delta-like 4 Induces Epigenetic Regulation of Treg Cell Differentiation and Function in Viral Infection. Mucosal Immunol..

[168] Batah S.S., Fabro A.T. (2021). Pulmonary Pathology of ARDS in COVID-19: A Pathological Review for Clinicians. Respir. Med..

[169] Xu G., Qi F., Li H., Yang Q., Wang H., Wang X., Liu X., Zhao J., Liao X., Liu Y. (2020). The Differential Immune Responses to COVID-19 in Peripheral and Lung Revealed by Single-Cell RNA Sequencing. Cell Discov..

[170] Alahyari S., Rajaeinejad M., Jalaeikhoo H., Amani D. (2022). Regulatory T Cells in Immunopathogenesis and Severity of COVID-19: A Systematic Review. Arch. Iran. Med..

